# Immunotherapy-Based Targeting and Elimination of Leukemic Stem Cells in AML and CML

**DOI:** 10.3390/ijms20174233

**Published:** 2019-08-29

**Authors:** Peter Valent, Irina Sadovnik, Gregor Eisenwort, Karin Bauer, Harald Herrmann, Karoline V. Gleixner, Axel Schulenburg, Werner Rabitsch, Wolfgang R. Sperr, Dominik Wolf

**Affiliations:** 1 Department of Internal Medicine I, Division of Hematology & Hemostaseology, Medical University of Vienna, 1090 Vienna, Austria; 2 Ludwig Boltzmann Institute for Hematology & Oncology, Medical University of Vienna, 1090 Vienna, Austria; 3 Department of Radiotherapy, Medical University of Vienna, 1090 Vienna, Austria; 4 Division of Blood and Bone Marrow Transplantation, Department of Internal Medicine I, Medical University of Vienna, 1090 Vienna, Austria; 5 Department of Internal Medicine V (Hematology & Oncology), Medical University of Innsbruck, 1090 Innsbruck, Austria; 6 Medical Clinic 3, Oncology, Hematology, Immunoncology & Rheumatology, University Clinic Bonn (UKB), 53127 Bonn, Germany

**Keywords:** AML, CML, leukemic stem cells, CAR-T cell therapy, bispecific antibodies, precision medicine

## Abstract

The concept of leukemic stem cells (LSC) has been developed with the idea to explain the clonal hierarchies and architectures in leukemia, and the more or less curative anti-neoplastic effects of various targeted drugs. It is now widely accepted that curative therapies must have the potential to eliminate or completely suppress LSC, as only these cells can restore and propagate the malignancy for unlimited time periods. Since LSC represent a minor cell fraction in the leukemic clone, little is known about their properties and target expression profiles. Over the past few years, several cell-specific immunotherapy concepts have been developed, including new generations of cell-targeting antibodies, antibody–toxin conjugates, bispecific antibodies, and CAR-T cell-based strategies. Whereas such concepts have been translated and may improve outcomes of therapy in certain lymphoid neoplasms and a few other malignancies, only little is known about immunological targets that are clinically relevant and can be employed to establish such therapies in myeloid neoplasms. In the current article, we provide an overview of the immunologically relevant molecular targets expressed on LSC in patients with acute myeloid leukemia (AML) and chronic myeloid leukemia (CML). In addition, we discuss the current status of antibody-based therapies in these malignancies, their mode of action, and successful examples from the field.

## 1. Introduction

Acute myeloid leukemia (AML) and chronic myeloid leukemia (CML) are stem cell-derived, life-threatening, hematopoietic neoplasms that are characterized by an uncontrolled expansion of myeloid progenitor cells exhibiting a more or less severe maturation defect. The clinical course, prognosis, and progression patterns vary among patients, depending on the genetic background, age, the sub-type and phase of the disease, cytogenetic and molecular features, and the patient’s response to initial anti-leukemic (induction) therapy [1,2,3,4,5,6,7,8].

Whereas in CML the disease is characterized by the pathognomonic *BCR-ABL1* oncogene, the molecular landscapes in AML are complex and involve several different oncogenes and a plethora of somatic mutations [4,5,6,7,8,9]. By applying intensive chemotherapy and oncogenic driver-specific drugs in AML and BCR-ABL1-targeting compounds in CML, a majority of all patients achieve remission, and in many cases, long-term disease-free survival is achieved [1,2,3,4,5,10,11,12,13]. However, not all patients have a good response to such therapy, or they relapse after having achieved remission. In patients with multi-resistant disease, hematopoietic stem cell transplantation (HSCT) is usually recommended, but the procedure can only be performed in a limited number of ‘young’ and fit patients, and carries an inherent mortality risk. As a result, research in AML is currently focusing on new molecular targets and the establishment of more potent drug therapies, including targeted drugs and immunotherapies.

The basic theory of leukemic stem cells (LSC) has been created with the intention to explain cellular and molecular hierarchies and to improve anti-neoplastic treatment through the eradication of disease-initiating and disease-propagating cells [14,15,16,17,18,19,20,21,22]. The concept of LSC is based on the hypothesis that the leukemic clone and sub-clonal evolution are organized in a cellular hierarchy, with (a) more mature leukemic cells that disappear (through apoptosis) after a certain number of cell divisions, and (b) LSC that can augment the bulk population of leukemic cells indefinitely by their unrestricted (unlimited) self-renewing and long-term proliferative abilities [14,15,16,17,18,19,20,21,22]. In the chronic phase of BCR-ABL1^+^ CML and in some AML variants, LSC were reported to reside in a CD34^+^/CD38^−^ subset of the leukemic clone [14,15,16,17,18,19,20]. However, depending on the molecular background and the phase of the disease, at least some LSC may also be detected within a CD34^+^/CD38^+^ subset of leukemic cells, or sometimes even in a CD34-negative cell population [23,24,25].

Based on their disease-initiating and disease-propagating capacity, LSC are regarded as a major, clinically relevant therapeutic cell target, and numerous studies have been conducted with the goal of identifying new molecular targets in these cells [17,18,19,20,21,22,26,27,28,29]. Of special interest are specific cell surface antigens that can be employed to develop disease-eradicating immunotherapies such as antibody-based or CAR-T cell therapies. However, only a few clinically relevant cell surface targets that are expressed specifically on LSC, but not on normal bone marrow (BM) stem cells, have been identified.

In the current article, we review the cell surface antigens that are expressed preferentially or even specifically on LSC in AML and/or CML, and thus represent potential targets for immunotherapies. In addition, we provide an overview of treatment concepts that have been or are currently being developed based on antigen expression and function in leukemic (stem) cells. Moreover, we discuss the current status of antibody-based therapies in AML. Finally, we discuss future developments in the field, and how LSC-targeting immunotherapies can be translated into clinical application.

## 2. Phenotype of LSC in AML and CML

The classical approach to demonstrate self-renewing and long-term disease-propagating abilities of LSC in vivo is to transplant leukemic cells into immunocompromised mice. Earlier studies employed severe combined immunodeficiency (SCID) mice or non-obese SCID (NOD/SCID) mice for long-term engraftment studies [14,15,16]. In these initial studies, the NOD/SCID mouse (long-term)-repopulating LSC in AML and CML were found to reside preferentially in a CD34^+^/CD38^−^ subset of the leukemic clone [14,15,16,30]. Therefore, most data on LSC refer to these cells. However, it soon turned out that the residual immune system of NOD/SCID mice can eliminate CD38^+^ AML cells, and that complete immunosuppression enables CD38^+^ AML LSC to produce long-term engraftment in NOD/SCID mice [23]. Therefore, highly immunocompromised (and thus more permissive) mouse strains were employed and soon accepted as a new standard model of xenotransplantation in AML. One of the most frequently used strains is NSG, a NOD/SCID mouse model lacking a functional interleukin-2 receptor gamma chain. In most AML variants, the NSG mouse-repopulating AML LSC reside in both the CD34^+^/CD38^−^ and CD34^+^/CD38^+^ fraction of the leukemic clone [23]. Similarly, in the blast phase of Ph^+^ CML, LSC appear to express CD38 [31], and the same holds true for Ph^+^ and Ph^−^ acute lymphoblastic leukemia (ALL) [32]. In other words, in acute leukemia models, LSC often reside in a ‘progenitor cell-like’, CD38^+^, fraction of the leukemic clone. Another issue is the molecular and phenotypic complexity and the related sub-clone formation in myeloid leukemias, including AML [22,25,33]. Finally, in certain forms of leukemia, some LSC may even reside in a CD34-negative compartment of the clone [24,25].

Overall, very little is known about the specific phenotypes of AML LSC. The only proven fact is that in most AML variants, LSC usually reside within the CD34^+^ compartment of the clone. Therefore, a target antigen can only be regarded as being displayed by AML LSC when the antigen is homogeneously expressed on all CD34^+^/CD38^−^ and all CD34^+^/CD38^+^ cells in the leukemic sample. Cell surface antigens that fulfill these criteria in several AML variants (most cases) include, among others, CD33 (Siglec-3), CD44 (Hermes), CD45 (common leukocyte antigen), CD47 (integrin associated protein = IAP) and CD123 (interleukin-3 receptor alpha-chain = IL-3RA) (Table 1) [34,35,36,37,38,39,40,41]. In about half of the patients with AML, both LSC-containing fractions (CD34^+^/CD38^−^ and CD34^+^/CD38^+^ cells) express C-type lectin protein-1, CLL-1 (CD371) [26]. This antigen is not expressed on normal CD34^+^/CD38^−^ stem cells [26]. Other cell surface antigens that are expressed preferentially or even selectively on CD34^+^/CD38^−^ AML LSC, but not (or only at trace amounts) on normal stem cells, include CD9, CD25, CD93, CD96, HLA-DR, and interleukin-1 receptor accessory protein (IL-1RAP) (Table 1) [35,37,40,41,42,43,44,45].

**Figure 1:**
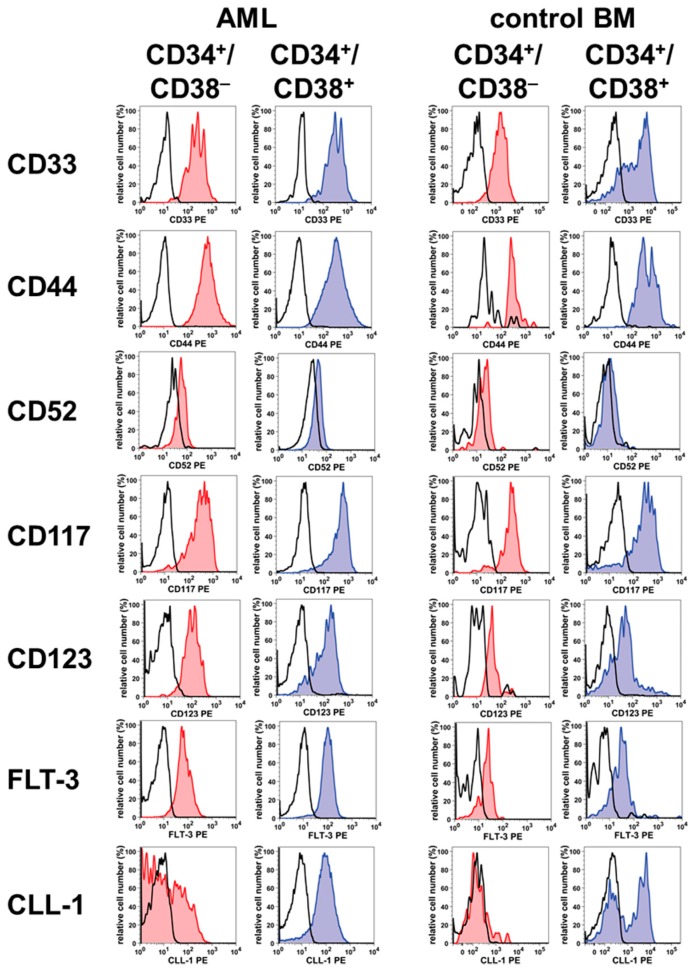
Examples of expression of cell surface target antigens on CD34^+^ stem cells in normal bone marrow (NBM) and patients with acute myeloid leukemia (AML). Target expression on aspirated CD34^+^/CD38^−^ cells (left panels, red histograms) and CD34^+^/CD38^+^ BM cells (right panels, blue histograms) was determined by fluorochrome-conjugated antibodies (as depicted) and multi-color flow cytometry. Normal/reactive BM (NBM) was obtained from lymphoma patients without BM involvement or was purchased, and leukemic BM was obtained from three patients with AML. All patients gave written informed consent before BM aspiration was performed. The study was approved by the ethics committee of the Medical University of Vienna. Reactivity of the test antibodies (CD34^+^/CD38^−^ stem cells: red histograms; CD34^+^/CD38^+^ stem/progenitor cells: blue histograms) was assessed on a FACSCantoII (BD Biosciences). Antibody reactivity was controlled by isotype-matched control antibodies (open black histograms). Flow cytometry data were analyzed using FlowJo 8.8.7 software (Flowjo).

CD34^+^/CD38^−^ AML LSC express higher levels of CD33 and CD123 compared to normal CD34^+^/CD38^−^ BM stem cells, but the therapeutic window is rather small. In other words, at least some normal stem cells may also be eliminated by intensive treatment with CD33-targeted drugs or CD123-targeted drugs. Other surface targets such as CD44 or CD52 (Campath-1) are expressed abundantly on normal stem cells as well as AML LSC [35,40,46] (Table 1, Figure 1). In most patients with AML, the CD34^+^/CD38^−^ LSC do not display CD26 (dipeptidyl-peptidase-IV = DPPIV) and CD90 (Thy-1) [29,47].

In chronic phase CML, LSC are considered to reside preferentially (if not strictly) in a CD34^+^/CD38^−^ population of the leukemic clone [28,29,30]. These cells exhibit CD25 (IL-2RA), CD26, and IL-1RAP in an aberrant manner in almost all cases (Table 1) [29,48,49,50,51,52]. In addition, CML LSC aberrantly display surface CD56 and CD93 in most cases (Table 1). CML LSC also exhibit higher levels of CD9, CD33, CD123, and HLA-DR compared to normal BM stem cells [29,48] (Table 1). In most patients with chronic phase CML, LSC do not express CLL-1 [29] (Table 1). Similar to AML LSC, CML LSC display a number of potential cell surface targets, including CD33, CD44, and CD123 [35,53,54,55,56]. In chronic phase CML, CD33 is homogenously expressed on most or all LSC, suggesting that targeting this surface receptor may result in LSC depletion [54].

An interesting aspect is that AML LSC and CML LSC also display various cytokine receptors, including the IL-2R alpha chain CD25, granulocyte colony-stimulating factor (G-CSF) receptor (CD114), stem cell factor receptor (SCFR = KIT, CD117), IL-3RA (CD123), and FLT3 (CD135) (Table 1) [34,35,49]. In a subset of patients, LSC also express macrophage colony-stimulating factor receptor (M-CSFR = CD115), granulocyte/macrophage colony-stimulating factor (GM-CSF) receptor alpha chain (GM-CSFRA = CD116) and/or insulin-like growth factor-1 receptor (IGF-1R = CD221) [35,49,57,58,59,60]. However, cytokine receptor expression on LSC may be low and hardly detectable by flow cytometry, even if receptor expression is sufficient to mediate cytokine effects in these cells.

**Figure 2:**
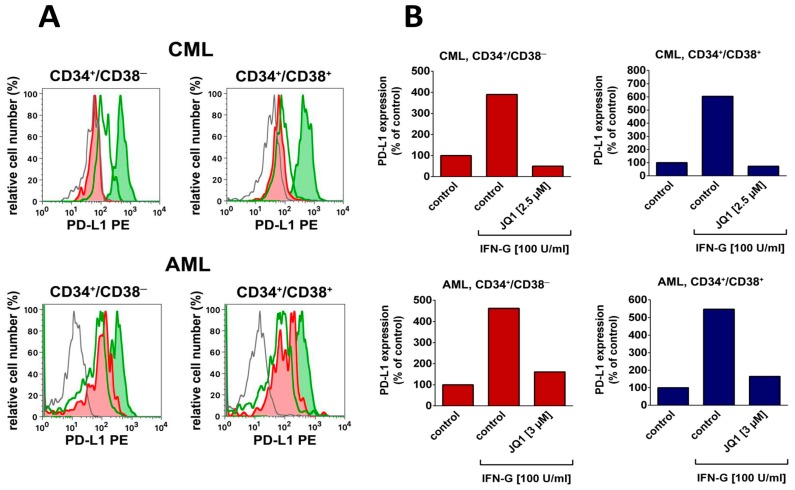
Expression of PD-L1 on leukemic stem cells and regulation by the BRD4/MYC blocker JQ1.A: Mononuclear cells (MNC) were obtained from the bone marrow of a patient with chronic leukemia (CML, upper panel) and from the blood of a patient with acute myeloid leukemia (AML FAB M4, lower panel). Both patients gave written informed consent before BM aspiration was performed. The study was approved by the ethics committee of the Medical University of Vienna. MNC were incubated in control medium (open green histogram), recombinant IFN-G (100 U/mL; green histogram) or in a combination of interferon γ (IFN-G) (100 U/mL) and 3 μM of JQ1 (AML) or IFN-G (100 U/mL) and 2.5 µM of JQ1 (CML) (red histograms) for 24 h at 37 °C. Then, the expression of PD-L1 on CD45^+^/CD34^+^/CD38^─^ LSC (left panels) and CD45^+^/CD34^+^/CD38^+^ stem and progenitor cells (right panels) was measured by a monoclonal antibody against PD-L1 on a FACSCalibur (BD Biosciences). The isotype-matched control antibody is shown as a grey histogram. B: shows the IFN-G-induced upregulation of PD-L1 (compared to medium control = control) on LSC (red bars, left panels) and on CD34^+^/CD38^+^ leukemic progenitors (blue bars, right panels) as a bar diagram in one patient with chronic phase CML (upper panels) and one patient with AML (FAB M4, lower panels) and the effects of JQ1 (3 µM) on IFN-G-induced upregulation of PD-L1 in these cells. FAB, French–American–British cooperation study group classification.

Finally, LSC in AML and CML display various immune checkpoint antigens, including the key checkpoint target PD-L1 (CD274) and the ‘don’t eat me’ receptor IAP (CD47) (Table 1) [61,62,63]. Whereas CD47 is expressed abundantly on LSC in a constitutive manner in most patients with AML and most with CML, the expression of PD-L1 is often weak or absent on LSC in these malignancies. However, the expression of PD-L1 can usually be induced or augmented substantially on LSC by exposure to interferon γ (IFN-G) (Figure 2, Table 1).

## 3. Targeting LSC with Antibody-Based Drugs

Whereas in lymphoid neoplasms, a number of different antibody-based drugs are available and are effective in eliminating neoplastic cells when applied together with chemotherapy, only a few antibody-based drugs are available for myeloid neoplasms (Table 2) [64,65,66]. This may be because compared to lymphatic neoplasms, it is more difficult to identify robust targets that are expressed preferentially or selectively on neoplastic stem cells and progenitor cells in myeloid neoplasms, but are not expressed (or only expressed at trace amounts) on normal myeloid stem cells. In fact, normal stem cells usually express many of the classical myeloid antigens on their surface, including CD13, CD33, CD44, CD47, CD52, CD117, and CD123. However, compared to LSC, expression levels on normal bone marrow stem cells are often significantly lower, thereby providing a small, albeit often suitable, therapeutic window [26,34,35,36,37,38,39,40,41,42,43,44,45]. One example is CD33 (Siglec-3) (Table 2). In fact, CD33 was soon identified as a potential target receptor displayed by AML LSC, and a number of previous and recent studies have shown that this antigen is a clinically useful therapeutic target [35,36,67,68,69,70,71,72].

Gemtuzumab ozogamicin (GO) is a humanized anti-CD33 antibody conjugated to a cytotoxic, calicheamicin-type drug through a bifunctional chemical linker [64,65,66]. Previous studies have shown that treatment with GO is effective in chemotherapy-refractory and relapsed AML. Subsequently, the drug was approved by the FDA in 2000. However, in post-marketing studies, the clinical benefit of GO could not be confirmed in relapsed AML [73]. Moreover, veno-occlusive liver disease and prolonged cytopenia (neutropenia and thrombocytopenia) were observed after repeated exposure to GO (as adjunct to chemotherapy) [67,68,69,70,71,72,73]. As a result, Pfizer withdrew GO (voluntarily) from the market in 2010, which may have been a premature decision.

**Figure 3:**
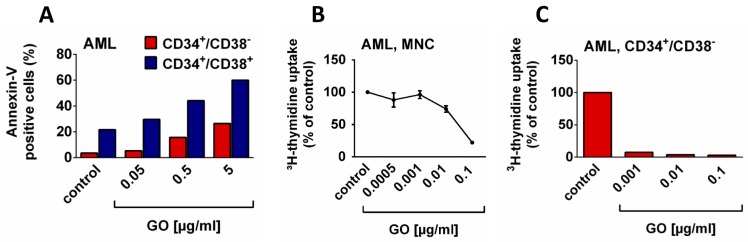
Effects of gemtuzumab ozogamicin (GO) on the growth and survival of leukemic (stem) cells in patients with acute myeloid leukemia (AML). A: AML cells from a patient with FAB M4 were cultured in the presence of control medium or medium supplemented with GO (0.05–5 µg/mL) for 48 h at 37 °C. Then, CD34^+^/CD38^−^ leukemic stem cells (LSC; red bars) and CD34^+^/CD38^+^ stem and progenitor cells (blue bars) were analyzed by multi-color flow cytometry. The percentage of apoptotic cells (in LSC/progenitors) was determined by Annexin-V and DAPI staining. Flow cytometry was performed on a FACSCanto (BD Biosciences). B: AML blasts (FAB M1) were cultured in control medium or medium supplemented with GO (0.0005–0.1 µg/mL) for 48 h at 37 °C. After incubation, ^3^H-thymidine uptake was measured. Results are expressed as a percentage of control and represent the mean ±S.D. of triplicates. C: CD34^+^/CD38^−^ stem cells from a patient with AML M4 were purified by cell sorting. Then, cells were cultured in control medium or in medium containing various concentrations of GO (0.001–0.1 µg/mL) for 48 h at 37 °C. After incubation, ^3^H-thymidine uptake was measured. Results are expressed as the percent of control in one experiment. All the patients gave written informed consent before BM cells were stored and analyzed. The study was approved by the ethics committee of the Medical University of Vienna. FAB, French–American–British cooperation study group classification.

More recent data have shown that therapy with lower and thus less toxic GO doses improves overall survival (OS) in AML patients with favorable cytogenetics, but is less effective in patients with poor cytogenetic features [74,75,76]. In a meta-analysis calculating the outcomes in five controlled randomized trials, GO was found to reduce the risk of relapse and to improved OS in patients with favorable cytogenetics as well as in those with intermediate cytogenetics [76]. Moreover, GO has recently been described to improve outcomes in older AML patients in two Phase III trials [77]. In all studies, when tested, the clinical benefit was preferentially seen in patients in whom AML blasts (CD34^+^ cells) display CD33 [78]. Correspondingly, GO induces apoptosis and growth arrest in AML LSC [54] (Figure 3). In 2017, the FDA approved GO for the treatment of patients with newly diagnosed AML as adjunct to standard induction polychemotherapy [79].

During the past few years, several attempts have been made to develop additional and more potent CD33-targeting antibody-based drugs. SGN-CD33A (vadastuximab talirine) is a novel CD33-targeted agent that contains a humanized anti-CD33 antibody with engineered cysteine residues, conjugated to a synthetic DNA-damaging agent (pyrrolobenzodiazepine dimer) through a protease-sensitive linker [80,81]. The antibody-conjugate is endocytosed by CD33^+^ AML cells and, once released, the DNA cross-linker delivers major cytotoxic effects [80]. Apparently, SGN-CD33A is a more potent agent against AML blasts compared to GO [80]. The drug exerts growth-inhibitory and apoptosis-inducing effects on chemotherapy-resistant AML blasts [81,82]. SGN-CD33A may be more effective than GO for several reasons. First, SGN-CD33A benefits from a novel linker technology that enables uniform drug loading of the antibody molecules. In addition, unlike GO, the DNA-damaging agent (a pyrrolobenzodiazepine dimer) is considered to deliver cytotoxicity in AML cells independent of multidrug resistance (MDR) antigens, such as MDR-1. Initially, it was also described that vadastuximab talirine is well tolerated in most patients when applied as a single agent [82]. In addition, the drug induced remission in patients with chemotherapy-refractory AML [83]. However, the therapeutic window regarding stem cell suppression was small, and when combined with cytotoxic drugs and polychemotherapy, hematologic toxicity was substantial and led to infection-related death, finally leading to the discontinuation of the Phase III trial and stopping of drug development [83].

A number of strategies have been developed to target the IL-3RA (CD123) on AML blasts and AML LSC. One promising strategy is to conjugate cytotoxic agents to IL-3. One such compound is the diphtheria toxin DT_388_/IL-3 fusion protein. This conjugate consists of the catalytic and translocation domains of diphtheria toxin coupled to human IL-3 [84,85,86]. A variant of this toxin conjugate (DT_388_ IL-3[K116W]) was obtained by fusing the diphtheria toxin with a modified IL-3 protein, which resulted in a stronger binding of IL-3 to the IL-3 receptor [87]. On a molar basis, DT_388_ IL-3[K116W] is more active than DT_388_ IL-3 in destroying leukemic cells [87]. Nevertheless, both DT_388_ IL-3 and DT_388_ IL-3[K116W] induce cytotoxic effects on IL-3R^+^ cells in vitro and in vivo. The extent of cytotoxicity induced by these compounds is directly correlated with the level of IL-3R expressed on the surface of AML blasts [58,85]. Preclinical studies have shown that DT_388_ IL-3 doses up to 100 μg/kg are well tolerated [86]. Based on these data, the related IL-3-toxin fusion protein SL-401 (tagraxofusp) was developed and evaluated in a Phase I clinical trial in heavily pre-treated AML patients (NCT 02270463): in a group of 70 AML patients, two complete responses and five partial responses were seen [87]. In a few other patients, stable disease was obtained and observed for more than one year, suggesting that some AML LSC had been eliminated [88]. Other studies have shown that tagraxofusp is active against LSC in patients with CML [89]. However, it remains unknown whether tagraxofusp will be further developed in the context of AML or CML [90]. More recent data suggest that tagraxofusp is an extremely active agent in plasmacytoid dendritic cell neoplasms [90,91], and in 2018, the drug received approval for this indication by the FDA [92].

Another principal way to target the IL-3R is to apply antibodies that bind to IL-3R with high affinity and thereby block the binding of IL-3 to this receptor. The 7G3 antibody binds to the N-terminal domain of the human IL-3R alpha chain (CD123) and acts as potent IL-3R antagonist [93]. 7G3 exerted potent inhibitory effects in vitro and in vivo on the growth of AML cells, including LSC, whereas its inhibitory effects on normal hematopoietic stem cells were reported to be minor if not negligible [93]. The subsequent humanization of 7G3 and its engineering (for optimal antibody-dependent cytotoxicity) resulted in the development of the CSL362 antibody [94]. CSL362 was found to be an effective agent that can inhibit the in vitro growth of CD123^+^ leukemic AML cells, including LSC [94]. However, when tested in a Phase I study in patients with chemotherapy-refractory AML, CSL362 did not exert sustained anti-leukemic activity in a majority of the patients examined [95].

There are also other surface targets that are expressed on AML LSC and are considered suitable for antibody-based treatment and clinical application in patients. These include, among others, CD26, CD44, CD45, CD47, CD56, and CD157 [96,97,98,99,100,101,102]. However, although these targets are all expressed on AML LSC (CD44, CD47, CD157) and/or CML LSC (CD26, CD44, CD47, CD56), clinical application, when tested in AML, resulted only in minor or transient effects on neoplastic cells [99,100,102].

Finally, several radiolabeled antibodies have been developed and considered for use in AML. These include, among others, ^131^I-labeled, ^213^Bi-labeled, or ^225^Ac-labeled anti-CD33 or anti-CD45 antibodies, and ^188^Rhe-labeled anti-CD66 antibodies [103,104,105,106,107]. However, most of these antibodies exert major hematologic toxicity with substantial or even long-term cytopenia (aplasia), which can be explained by the cross-radiation effects on surrounding normal hematopoietic stem cells and by the accumulation of these antibodies in hematopoietic tissues, including the spleen and BM [103,104,105,106,107]. Therefore, these antibodies are mostly applied in combination with an HSCT approach [103,104,105,106,107]. Whether some of these antibody-based therapies when added to conventional conditioning regimens are helpful or even superior in the preparation for HSCT in AML patients remains at present unknown.

## 4. Targeting LSC Using Drugs Directed against Immune Checkpoint Molecules

During the past few years, several therapeutic concepts have been developed with the aim to overcome immune checkpoint-mediated, immunologic resistance of neoplastic (stem) cells [107,108,109,110,111,112,113]. Major checkpoint molecules that are detectable on cancer/leukemic cells include CD28, CTLA4, PD-L1, PD-L2, and TIM3. PD-L1 is a checkpoint molecule that has been studied extensively in solid tumors, and the concept of blocking its activity using specific antibodies has recently been translated into clinical application also in myeloid neoplasms [107,108,109,110,112]. In fact, several PD1-targeting or PD-L1-targeting antibody-based drugs are available, and have been shown to block PD1–PD-L1 interaction, and thereby the immune checkpoint-mediated resistance of neoplastic cells in patients with melanoma, Hodgkin disease, and other neoplasms [107,108,109]. More recently, preclinical and clinical studies employing PD1-targeting or PD-L1-targeting antibodies have also been conducted in myeloid neoplasms, including AML [110,111,112,113]. However, responses to these antibodies turned out to be variable and often transient if at all measurable [110,111,112,113]. Therefore, antibodies targeting PD1–PD-L1 interaction have been combined with cytoreductive or hypomethylating agents in myelodysplastic syndromes (MDS) and AML [110,111,112,113,114]. One important aspect here is that LSC in AML, CML, and MDS do not express PD-L1 in a constitutive manner in all patients (Table 1). Rather, in most patients, LSC and more mature leukemic cells only express substantial amounts of PD-L1 in these malignancies under certain conditions. In general, three mechanisms lead to the expression of PD–L1 on LSC. One is the cytokine storm, with IFN-G being the strongest signal (Figure 2) [61,115,116]. A second mechanism relates to the oncogene-dependent expression of PD-L1: notably, several oncoproteins and related pathways, such as JAK-STAT (a key driver being JAK2 V617F) or MYC-related pathways can upregulate PD-L1 expression on LSC [61,117,118]. Finally, certain drugs, such as the hypomethylating agents can increase the expression of PD-L1 on leukemic cells, including AML blasts and AML LSC [119]. Therefore, combinations of PD-L1 or PD1 inhibitors with hypomethylating agents are currently being tested in clinical trials in patients with AML [110,111,112,113,114]. However, the hypomethylation-induced upregulation of PD-L1 is not seen in AML LSC in all patients (P.V., unpublished observation).

During the past few years, the molecular mechanisms underlying the cytokine-induced expression of PD-L1 on LSC have been examined. In these studies, the BRD4–MYC axis and the JAK-STAT pathway have been identified as major drivers of PD-L1 expression [61,117,118]. In line with this concept, the BRD4/MYC-targeting drug JQ1 inhibits the IFN-G-induced expression of PD-L1 on LSC in patients with AML and CML (Figure 2) [61]. Whether the targeting of PD-L1 expression by BRD4/MYC blockers is relevant clinically remains at present unknown. It is worth noting in this regard that most BRD4/MYC blockers and especially the BRD4 degraders also exhibit strong direct anti-neoplastic effects on AML LSC [120,121], and may overcome multiple forms of LSC resistance in AML and CML (P.V., unpublished observation).

Another interesting checkpoint molecule is CTLA4. Although it remains unknown whether this immune checkpoint molecule plays a role in LSC resistance in AML, it is worth mentioning that CTLA4 blockade may be useful in the post-HSCT setting in hematologic malignancies, including AML [122,123].

Finally, TIM-3 is a promising new checkpoint antigen in the AML context. In fact, TIM-3 and its ligand Galectin-9 have been shown to constitute an autocrine loop that is critical for the survival of AML LSC [124]. Moreover, AML patients have increased Galectin-9 levels, which may augment stem cell signatures and LSC renewal via TIM-3. Interestingly, Galectin-9/TIM-3 expression is upregulated in patients failing chemotherapy [125], and is associated with central memory and memory stem T cell exhaustion in AML patients with disease relapse after HSCT [126]. The clinical value of blocking Galactin-9/TIM-3 interaction alone or in combination with other targeted drugs is currently being explored in clinical trials in high-risk MDS and AML.

## 5. Targeting of LSC by Bispecific Antibodies

Bispecific antibodies are engineered drugs recognizing two different epitopes or two different antigens. These agents have several advantages over conventional targeted antibody constructs and monospecific toxin conjugates. First, bispecific antibodies can be engineered to recruit T cells, natural killer (NK) cells, or other relevant cells of the immune system [127,128,129,130]. Moreover, these drugs can be designed to recognize and block multiple surface targets on LSC, including classical molecular targets or key checkpoint molecules, such as PD-L1, PD-1, or CD47. Finally, although receptor internalization is a validated mode of action of some bispecific antibodies, it is not a general prerequisite for the functionality of bispecific antibodies in AML [127,128,129,130].

Among others, there are two types of fragment-based bispecific antibodies used in cancer research: bispecific T cell engagers (BiTEs) are recombinant fusion antibodies designed by utilizing two single-chain variable fragments (scFvs) tandemly arranged on a polypeptide chain. Dual affinity retargeting (DART) antibodies are diabodies consisting of heavy-chain and light-chain variable domains of two antigen-binding specificities linked to two distinct polypeptide chains that can heterodimerize and are connected with a cystine bond.

One of the first bispecific antibodies used in applied hematology was blinatumomab. This BiTE binds CD3 and CD19, and is able to provoke the T cell-mediated killing of B cells [131,132,133]. After first successful clinical trials had been conducted and published, blinatumomab received accelerated approval from the FDA for the treatment of either minimal residual disease-positive or relapsed/refractory B-lineage ALL [134].

Subsequently, several attempts have been made to develop similar agents for the treatment of AML. Indeed, a number of BiTEs, DARTs, and tandem diabodies are in preclinical and clinical development for AML [131,132,133]. The antibodies that are currently being tested are directed against three key target antigens on LSC, namely CD33, CD123, and CLEC12A/CLL-1 (CD371) [131,132,133]. A detailed description of the preclinical and clinical effects of bispecific antibodies in AML is beyond the scope of this article. We refer the reader to the available literature [131,132,133,134,135,136,137,138,139,140,141,142]. In many instances, clinical trials are still ongoing. The final results of these ongoing studies in AML will elucidate the real potential and clinical perspective of these agents in the AML context. A common toxicity associated with T cell redirecting therapy (including bispecific antibodies) is a cytokine release syndrome (CRS) [131,132,133]. Other side effects are cytopenia and liver toxicity. However, in general, BiTEs and DARTs are relatively well tolerated with an acceptable toxicity profile.

Bispecific CD33-targeting antibodies tested in clinical trials in patients with AML include, among others, the CD33/CD3-specific drugs AG330 and AMG673, the tetravalent CD33/CD3 tandem diabody construct AMV564, and the humanized single-chain bispecific CD33/CD3 antibody GEM333 (Table 3) [135,136,137,138,139].

Targeted antibodies against CD123 currently tested in AML include, among others, the CD123/CD3 DART flotetuzumab, the CD123/CD3-engaging IgG4 DuoBody JNJ-63709178, and the CD123-targeting X-mAb XmAb14045 (Table 3) [140,141,142]. The human full-length IgG1 bispecific antibody MCLA-117 targeting CLL-1 is also currently tested in AML (Table 3) [143]. Flotetuzumab showed measurable anti-leukemic effects in 8/14 patients with chemotherapy-refractory AML (57%), and two of these patients achieved complete remission [139]. This drug is currently tested in a Phase I dose-expansion study. Overall, however, little is known about the clinical efficacy and toxicity of the above-mentioned BiTEs, DARTs, and other immune-cell-recruiting agents in AML. A number of additional bispecific and other multi-targeting antibodies with diverse specificities and multiple mechanisms of action are currently being developed in AML, with the hope to target disease-initiating LSC [143,144,145,146,147,148]. Whether these drugs will reach clinical application and help eradicate LSC remains to be determined in preclinical and clinical studies.

## 6. Targeting of LSC by Chimeric Antigen Receptor (CAR) Cell-Based Therapy

In the past few years, major efforts have been made to develop the adoptive transfer of immune cells expressing genetically engineered CAR (CAR-T or CAR-NK) in hematologic malignancies, including AML [149,150,151,152,153,154,155,156,157,158,159,160]. CARs consist of a polypeptide chain combining the extracellular antigen-binding site of an antibody, in generally a single-chain fragment of its variable region (scFv), to the intracellular CD3ζ chain that links the CAR to the signal cascade of the T cell receptor. CARs can be generated against every identified leukemic-associated antigen for which an antibody exists, and the genetic modification of T cells to express CAR can redirect them against leukemic (stem) cells in a non-HLA-restricted manner. A number of surface molecules expressed on AML LSC and CML LSC may serve as robust CAR-T cell-targeted antigens. These include, among others, CD33, CD44, CD123, CD135 (FLT3), CD371 (CLL-1), and Lewis Y (LeY) [75,149,150,151,152,153,154,155,156,157,158,159]. However, despite numerous preclinical studies, relatively few CAR-based approaches have been investigated in clinical trials in AML so far [150,159]. A summary of these trials is shown in Table 4. So far, one report of a patient with refractory/resistant AML treated with anti-CD33 CAR-T cells within a phase I trial (NCT01864902) was published [150]. This patient received a total of 1.12 × 10^9^ autologous T cells (38% CAR-transduced). Subsequently, the patient suffered from CRS as well as from prolonged pancytopenia (with blast cell clearance), and a relapse of disease was recorded nine weeks after CAR-T cell infusion [150]. In 2013, Ritchie et al. published a phase I study of autologous CAR anti-LeY T cell therapy in AML [159]. The infused CAR-T cells persisted for up to 10 months. Remarkably, grade 3 or 4 toxicity was not observed. Although three patients responded and even one cytogenetic remission was recorded, all patients relapsed after 1–23 months. Most other CAR-T cell therapies are in preclinical development or have just started to enter clinical application in phase I trials in AML (Table 4). Based on the encouraging preclinical data obtained with CAR-T cell therapies directed against CD33, CD123, CLL-1, and FLT3, there is some hope that these approaches will also work in vivo in patients with refractory AML.

However, there are a number of pitfalls and general issues to discuss when considering CAR-T cell approaches in AML. First, apart from logistic and financial obstacles, CAR-T cell therapy requires vast knowledge about the technology, practical issues, and technical details, and can therefore only be offered in specialized centers where AML is also a focus of research. Second, there are several side effects that have to be considered with CAR-T cell treatment in AML, including a tumor lysis syndrome, a CRS, prolonged cytopenia/aplasia, and other ‘on-target but off-tumor’ toxicities. Finally, current strategies in AML are not addressing the complexity of AML and AML LSC, but rather focus on only one or a few target structures. As a result, relapses are seen quite frequently. Therefore, the future of CAR-T cell therapy in AML may be to direct the CAR-T or CAR-NK approach against multiple LSC-specific targets that cover most AML sub-clones, but still spare normal myeloid stem cells. Another interesting approach is to apply CAR-NK cells instead of CAR-T cells (Table 4). Another question is when to apply CAR-T or CAR-NK cells in AML. Based on the toxicity profile and their ability to eliminate even residual dormant LSC, one proposed strategy is to use CAR cells in patients with minimal residual AML. In CML, no CAR-T or CAR-NK cell therapies have been developed so far. However, preclinical data obtained in mice suggest that CAR-T cells directed against IL-1RAP produce strong anti-leukemic effects on BCR-ABL1+ cells in vivo [160].

## 7. Targeting LSC by Employing NK Cells and/or T cells

Independent of therapy or lymph node infiltration, T cell and NK cell production and activity are substantially suppressed in patients with AML and other myeloid neoplasms. Therefore, multiple therapeutic strategies have been considered with the idea to restore or enhance T cell and/or NK cell numbers/activity and to direct effector cell activity against leukemic (stem) cells [161,162,163,164,165,166,167,168,169,170]. These strategies are summarized in Table 5. One approach is to expand allogeneic NK cells in vitro and apply these cells to patients together with HSCT in patients with AML [162,163,164]. Alloreactive NK cells are indeed known to exert cytotoxic effects on AML cells, to improve engraftment, and to boost graft versus leukemia (GVL) activity in AML patients receiving allogeneic HSCT [165,166,167,168]. In a recent phase I study, 21 patients with myeloid malignancies received haploidentical NK cells after conditioning with busulfan and fludarabine prior to HSCT from another donor [163]. No increase in the number of serious acute graft versus host disease (GVHD) events was reported. Although a clear survival benefit was not seen, a trend toward improved survival in NK cell-infused patients was found. Several studies with haploidentical NK cell infusion have been conducted in refractory/relapsed AML. In one study, eight patients with AML or MDS following prior HSCT received lymphodepletion followed by donor NK cell infusion and IL-2 [164]. Although one patient was driven into remission, no overall survival benefit was observed, and no donor NK cells were detected after infusion in these patients [164]. In another study, employing donor NK cell infusions on days +13 and +20 after allogeneic HSCT for refractory/relapsed AML, patients receiving larger numbers of alloreactive NK cells had an improved relapse-free survival [165]. Overall, the anti-AML effect of NK cells is well documented, but there is an ongoing discussion about the optimal way of activating these cells against AML LSC and how and when to apply NK cell infusions. An alternative strategy is to induce or promote antibody-mediated NK cell activity against AML (stem) cells. In fact, NK cells also exhibit antibody-dependent cellular cytotoxicity, and several antibodies have been applied to activate NK cells against AML cells. For example, an Fc-optimized CD133 antibody has successfully been used to activate CD16^+^ NK cells against AML cells and to provoke the killing of these cells while sparing normal hematopoietic stem cells [171]. Another approach tested recently was to prime NK cells against AML cells with an antibody against NKG2A, with the hope to induce the differentiation and activation of NK cells [172].

As mentioned before, several efforts have been made to engineer NK cells using the CAR technology, with the aim to augment their affinity and cytotoxicity against AML (stem) cells. Whether CAR-NK cells are better ‘CAR drivers’ than CAR-T cells is currently under discussion [173]. It also remains unknown whether a combined approach of boosting NK and T cells together in parallel to augment their anti-leukemic effects can elicit synergistic effects on AML LSC in patients.

Finally, a number of attempts have been made to prime NK cells with diverse cytokines to transform them into optimal killers that are capable of eradicating AML LSC. Cytokine-induced killer cells (CIK) are usually generated by exposing blood lymphocytes to IL-1, IL-2, or IL-15. In each case, CIK represent a heterogeneous population of killer cells expressing both T cell (CD3) and NK (CD56) cell markers. These cells have a broad range of anti-neoplastic activities, and are capable of exerting cytotoxicity against leukemic cells in MCH-restricted and MHC-unrestricted fashions. Several preclinical studies and phase I trials employing CIK cells in AML have been conducted and shown the feasibility and safety of this approach [174,175,176,177,178]. In some of these patients, the efficacy of CIK cells could be demonstrated [174,175,176,177]. However, CIK cell infusions are not effective in all patients, and several questions remain concerning the optimal preparation of CIK in AML, the optimal indication of CIK cell therapy, and the ability of CIK cells to eradicate AML LSC. In one recent study, IL-15 was successfully applied as a CIK activator in patients with AML [177]. Another strategy, tested recently, is to combine CIK cells with a specific CAR-T cell approach [178]. In this study, CIK cells directed against AML cells were engineered to express a CD123 CAR via retroviral transduction. These CD123 CIK CAR-T cells exerted major anti-leukemic effects in vitro on AML cells exhibiting CD123 [178]. Whether these combined CIK-CAR-T therapies are able to attack or even eliminate AML LSC is currently being tested in clinical trials.

A quite old strategy is to boost anti-leukemic NK cell and T cell effects by inducing NK cell and T cell expansion and activation with in vivo administrated IL-2. In initial studies, no substantial effects were observed [179,180,181]. Later, it was described that responses of NK cells to IL-2 are blocked by the generation of radical oxygen species (ROS) [182]. In order to overcome the ROS blockage of the immune system, combinations of IL-2 and histamine (blocks production of ROS) were applied [183,184,185]. These studies were mostly performed in AML patients in complete remission (CR), and showed convincing results with a survival benefit for CR patients treated with IL-2+histamine [184,185]. Subsequently, the drug combination was approved as maintenance immunotherapy in AML by the European Medicines Agency (EMA) in 2008. More recent data suggest that IL-2+histamine therapy may be particularly efficacious in AML patients with normal karyotypes [186]. It has also been described that IL-2 and histamine can indeed mobilize the NK cell and T cell system in these patients [187,188]. Despite this effect, side effects are usually mild and tolerable.

However, several issues remain with IL-2+histamine as maintenance therapy. First, the patients and doctors need to be trained in detail in order to avoid immediate adverse reactions. Notably, both compounds, when injected too quickly, may provoke serious adverse events, including hypotension, tachycardia, and flush, or even anaphylaxis. Another problem is that it remains unknown whether IL-2+histamine needs to be administered life-long or only for a certain time period. Moreover, the immunological effects of IL-2+histamine remain insufficiently understood. For example, the drug also induces regulatory T cells, which are assumed to counteract AML-suppressing immune responses. Thus, biomarkers confirming the efficacy and the quality of responses to IL-2+histamine therapy are lacking, and it remains uncertain what cohorts of patients may indeed benefit clinically from this type of immunotherapy.

## 8. Targeting LSC by Suppressing or Promoting LSC Homing

Depending on the type of leukemia, LSC are either fixed to the stem cell niche and enjoy niche-mediated protection against various toxic agents including therapeutic drugs (such as normal stem cells) or are mobilized cells that are not only fixed to the niche but are also capable of redistributing easily from the BM into other organ sites, and thus into extramedullary niches [189,190,191]. In most AML variants, some or even most LSC may be protected by the BM niche, although some LSC may be mobilized cells and also infiltrate into other organs sites, especially in monoblastic leukemias. However, during progression, AML (stem) cells become more and more independent of niche protection, and many LSC may infiltrate into extramedullary organs.

In CML, the situation is different: here, most LSC may be mobilized cells that can traffic into other organs, such as the spleen and form local pools of LSC, thus promoting extramedullary myeloproliferation. Several different mechanisms may underlie stem cell mobilization in AML and CML. First, the loss of certain adhesion molecules may promote redistribution out of the niche [192,193,194]. Second, enzymes produced by granulocytes or endothelial cells can promote mobilization by degrading surface adhesion receptors or cytokines responsible for stem cell–niche interactions. A good example is CML: here, LSC display dipeptidyl-peptidase IV, DPPIV (CD26). This is an enzyme that degrades stroma cell-derived factor-1 (SDF-1), which is known to be essential for stem cell homing in BM niches [29]. As a result, CML LSC mobilize into the blood [29]. An unresolved question is whether the mobilization of LSC in AML and CML can support or would even block the drug-induced killing of LSC. Initial studies with the CXCR4-inhibitor plerixafor—a drug known to induce the mobilization of AML LSC—did not show convincing beneficial effects in AML patients [195,196]. Another strategy is to block DPPIV in CML with gliptins [29]. However, so far, no clinical trials with DPPIV inhibitors or CD26-blocking antibodies have been conducted. Another strategy is to block the extramedullary spread of AML LSC by applying antibodies directed against homing and invasion receptors such as CD44. Indeed, antibodies against CD44 were found to reduce leukemic expansion in vivo in a xenotransplantation model. However, this effect was not confirmed in clinical studies so far: for example, the effects of the humanized anti-CD44 antibody RG7356 was tested in a phase I trial in patients with refractory or relapsed AML [99]. Only one out of 44 patients achieved a complete response with incomplete platelet recovery; one patient achieved a partial response, and one experienced a stable disease with hematologic improvement [99]. All in all, neither the mobilization concepts proposed nor the invasion–receptor blockers were found to improve therapy in patients with AML.

## 9. Limitations of LSC-Targeting Immunotherapy in AML and CML: LSC Resistance

Based on their selective ability to propagate the disease for unlimited time periods, LSC are attractive therapeutic targets in AML and CML, and numerous attempts have been made to selectively kill these cells by applying specific therapies [14,15,16,17,18,19,20,21,22]. However, the LSC pool (in individual patients and overall) consists of heterogeneous populations of cells (sub-clones) with varying molecular expression profiles and different patterns of cell surface antigens [22,23,24,25,31,33,198,199,200,201]. Moreover, most of the sub-clones are small-sized, and thus are not detectable at first diagnosis, which is a phenomenon that is associated with the low proliferative capacity of pre-leukemic neoplastic stem cells [22,197,198,199,200,201]. However, the most important issue is that LSC and their pre-leukemic neoplastic stages are highly resistant against various drug therapies [17,18,19,20,21,22,198,199,200,201,202,203]. In particular, a number of different mechanisms underlie LSC resistance in AML and CML (Table 6). In general, LSC resistance can be divided into i) intrinsic resistance (common to all LSC sub-clones and often also shared with normal stem cells), ii) acquired resistance (due to somatic mutations, the loss of tumor suppressors, or the loss of other antigens), iii) niche-mediated resistance, and iv) immunological resistance (often associated with the expression of immune checkpoints on LSC). Intrinsic LSC resistance is often associated with stem cell dormancy and/or with drug-exporter (drug-pumping) molecules. This type of resistance can often be overcome by antibody-based (ADC-based) or immune cell-based therapy, unless the antibody-conjugated, cytostatic drug is exported by an active drug pump once it is released from the antibody complex. Otherwise, antibody-based or cell-based therapies are optimal strategies to overcome intrinsic LSC resistance. Acquired resistance may or may not be overcome by immunotherapy or cell-based therapies (Table 6). For example, many cell-based therapies overcome acquired mutation-induced resistance against tyrosine kinase inhibitors. On the other hand, acquired resistance may also lead to loss of the major molecular surface targets (e.g., the loss of CD33 during treatment with GO). Another form of resistance against immune surveillance is oncogene-induced or cytokine-induced expression of checkpoint molecules, such as PD-L1 [61,115,116,117,118]. There are also therapy-specific forms of LSC resistance that have to be considered. For example, apart from target loss, a decrease or loss of CAR-T cells and/or the development of autoantibodies are important mechanisms of resistance against CAR-T cell therapies (Table 6) [204,205]. Finally, the niche-mediated resistance of LSC plays an important role in many leukemic disorders, including AML and CML [189,190,191]. In summary, multiple forms of LSC resistance have to be addressed in AML and CML when considering the development of curative (LSC-eradicating) drug therapies, and even immunotherapy-based approaches may not be able to overcome all these resistance mechanisms. Therefore, we believe that immunotherapy concepts have to be combined with other LSC-targeting treatment concepts in order to improve the overall outcome and survival in these patients. The type of drug combination depends on the type and phase of disease, the expression of cell surface and cytoplasmic drug targets, the presence of resistance-inducing mechanisms, and also patient-related factors such as age.

## 10. Concluding Remarks and Outlook to the Future

From a historical point of view, the first therapy that combined LSC eradication with immunotherapy in myeloid neoplasms was HSCT, and this approach is still considered standard in eligible patients with relapsed or refractory AML and CML. Later, donor lymphocyte infusion therapy confirmed the critical role of the allogeneic immune system in these patients. However, only a few patients who are young and fit can undergo HSCT. During the past 10 years, more elegant ways to eradicate LSC and to mobilize the immune system against LSC have been developed. First, the phenotype and target expression profiles of LSC in AML and CML have been established. In addition, novel targeted treatment concepts have been designed, including new antibody-based therapies, checkpoint inhibition, CAR-T and CAR-NK cell strategies and T/NK-mobilizing approaches. More recently, profound attempts have been made to merge LSC-targeting and immunotherapy concepts using novel tools and agents, with the ultimate aim to eliminate most or even all LSC in AML and CML. However, the oncogenic machineries in LSC are complex and associated with multiple mechanisms of resistance. In addition, most of these therapies may provoke side effects, including prolonged cytopenia and CRS requiring special attention and management in highly specialized centers. In order to overcome LSC resistance and to reduce side effects, combination strategies have been considered and are currently being tested in clinical trials. Whether these approaches improve the treatment and the overall outcomes of patients with AML and CML remains to be determined.

## Figures and Tables

**Table 1:** Expression of potential therapeutic targets on CD34^+^/CD38^–^ and CD34^+^/CD38^+^ cells in acute myeloid leukemia (AML) and chronic myeloid leukemia (CML) and comparison to stem cells in normal bone marrow (NBM) *.

Antigen	CD	Antigen Expression on Stem/Progenitor Cells in **					
		NBM		AML		CML	
		CD34^+^/ CD38^–^	CD34^+^/ CD38^+^	CD34^+^/ CD38^–^	CD34^+^/ CD38^+^	CD34^+^/ CD38^–^	CD34^+^/ CD38^+^
B4	CD19	–	–	+/–	+/–	+/–	+/–
B1	CD20	–	–	–	–	–	–
FceRII	CD23	–	–	–	–	–	–
IL2RA	CD25	–	–	+/–	+/–	+	–/+
DPPIV	CD26	–	–	–/+	–/+	+	–/+
Ki-1	CD30	+/–	+/–	+/–	+/–	+	+
Siglec-3	CD33	+	+	+	+	+	+
Hermes	CD44	+	+	+	+	+	+
IAP	CD47	+	+	+	+	+	+
Campath1	CD52	+/–	+/–	+/–	+/–	+	–/+
NCAM	CD56	–	–	–	–	+	+
G-CSFR	CD114	+/–	+	+	+	+	+
KIT	CD117	+	+	+	+	+	+
IL3RA	CD123	+	+	+	+	+	+
PROM1	CD133	+	+	+	+	+	+/–
FLT3	CD135	+/–	+/–	+	+	+/–	+/–
CXCR4	CD184	+	+	+	+	+	+
PD-L1	CD274	+/−	+/−	+/− ***	+/− ***	+/− ***	+/− ***
CLL-1	CD371	–	+	+/–	+	–	+/–
IL-1RAP	n.c.	–	+/–	+/–	+	+	+

* Data refer to the available literature [26,27,28,29,33,34,35,36,37,38,39,40,41,42,43,44,45] and/or data obtained by multi-color flow cytometry in the labs of the authors. ** Score: +, strongly expressed on most or all cells; +/− weak expression on most cells or expressed on subsets of cells (10–50%); −/+, weak expression on cells or expressed on cells in a small subset of donors; −, not expressed on stem cells. *** PD-L1 expression can be induced or enhanced by exposure of CML/AML cells to interferon γ. Abbreviations: NBM, normal bone marrow; FceRII, Fc-epsilon receptor II; IL-2RA, interleukin-2 receptor alpha chain; DPPIV, dipeptidyl peptidase IV; IAP, integrin associated protein; NCAM, neural cell adhesion molecule; G-CSFR, granulocyte colony-stimulating factor receptor; PROM1, prominin-1; PD-L1, programmed cell death 1 ligand 1; CLL-1, C-type lectin-like molecule-1; IL-1RAP, interleukin-1 receptor accessory protein; n.c., not yet clustered.

**Table 2:** Overview (digest) of conventional antibody constructs and toxin conjugates that are directed against LSC targets and have been developed for the treatment of AML.

Target	Name of Agent	Type of Antibody	Development Stage
CD33	Gemtuzumab ozogamicin (mylotarg)	ADC	Approved for treatment of AML
CD33	SGN-CD33 (lintuzumab)	ADC	Phase III (+CT) completed
CD33	SGN-CD33A (vadastuximab talirine)	ADC	Discontinued (toxicity)
CD33	IMGN779 (CD33-DGN462)	ADC	Phase I completed
CD33	Lintuzumab-^90^Y	RADA	Phase I completed
CD33	Lintuzumab-^213^Bi	RADA	Phase I/II completed
CD33	Lintuzumab-^225^Ac	RADA	Phase I completed
CD45	Various radiolabeled antibodies combined with CT and HSCT	RADA	Phase I, I/II, or III completed/ongoing
CD123	CSL362	HmAb	Phase I completed
CD123	KHK2823	HmAb	Phase I, active, not recruiting
CD123	JNJ-56022473 (CSL362) (talacotuzumab)	HmAb *	Discontinued
CD123	SGN-CD123A	ADC	Phase I, terminated
CD123	IMGN632	ADC	Phase I, recruiting
CD123	SL-401 (tagraxofusp **)	TOX-C	Approved for treatment of plasmacytoid dendritic cell neoplasms
CD25	Denileukin diftitox ***	TOX-C	Marketing discontinued

* Talacotuzumab exhibits an engineered Fc region, which increases the binding affinity to Fc gamma receptors on NK cells, thereby promoting antibody-dependent cytotoxicity (ADCC). ** Tagraxofusp is a toxin conjugate (TOX-C) consisting of human interleukin-3 (IL-3) and a truncated diphtheria toxin. *** Denlileukin diftitox (ontak) was a TOX-C containing IL-2 and diphtheria toxin. Abbreviations: LSC, leukemic stem cells; AML, acute myeloid leukemia; ADC, antibody–drug conjugate; RADA, radiolabeled antibody; HmAb, humanized monoclonal antibody; CT, chemotherapy; HSCT, hematopoietic stem cell transplantation; NK: natural killer.

**Table 3:** Bispecific antibodies currently tested in clinical trials in AML.

Name of Agent	Type of Agent	Target	Effector *	Phase	NCT
AMG330	BiTE	CD33	CD3	I	NCT02520427
AMG673	BiTE	CD33	CD3	I	NCT03224819
AMV564	Tandem diabody	CD33	CD3	I	NCT03144245
GEM333	Single-chain diabody	CD33	CD3	I	NCT03516760
161533 **	TriKE	CD33	CD16	I/II	NCT03214666
MGD006 (flotetuzumab)	DART	CD123	CD3	I	NCT02152956
JNJ-63709178	DuoBody	CD123	CD3	I	NCT02715011
XmAb14045	X-mAb ***	CD123	CD3	I	NCT02730312
MCLA-117	Biclonics ****	CD371	CD3	I	NCT03038230

* Effector: Targeted molecule on the effector cells. ** 161533 is a CD33 x CD16 TriKE that contains an interleukin-15 (IL-15) cross-linker and thereby is considered to augment NK cell expansion and function and to correct NK cell dysfunction in AML. *** X-mAb are antibody constructs that include a bispecific Fc domain that serves as a scaffold for the two antigen-binding domains. **** Bispecific antibody that binds to CD3 to recruit T cells. Abbreviations: AML, acute myeloid leukemia; NCT, national clinical trial identifier; BiTE, bispecific T cell engagers; TriKE, tri-specific killer engager; DART, dual affinity retargeting antibody.

**Table 4:** Clinical trials employing CAR-T cells or CAR-NK cells in AML.

CAR Target	Effector Cell	Phase	Patients/Cells/ Indications	Country	NCT
Lewis Y	T	I	Myeloma, AML, MDS	Australia	NCT01716364
CD33	T	I	CD33+ AML	USA	NCT03126864
CD33	T	I	R/R AML	China	NCT02799680
CD33	T	I/II	R/R AML	China	NCT01864902
CD33	NK	I/II	R/R CD33+ AML	China	NCT02944162
CD123	T	I	CD123+ AML	China	NCT03585517
CD123	T	I	relapsed AML after HSCT	China	NCT03114670
CD123	T	I/II	R/R AML	China	NCT03556982
CD123	T	I	R/R AML	USA	NCT02623582
CD123	T	I	R/R AML and R/R blastic plasmacytoid DCN	USA	NCT02159495
CD123	T	I	R/R AML	China	NCT03672851
CD123	T	I	R/R AML	USA	NCT03766126
UCART 123	T	I	R/R AML and newly diagnosed high-risk AML	USA	NCT03190278
CD123/CLL-1	T	II/III	R/R AML	China	NCT03631576
CD33, CD38, CD56, CD117, CD123, CD34, Muc1	T	I	R/R AML, MDS, ALL	China	NCT03291444
CD33, CD38, CD56, CD117, CD123, CD133, CD34 or Muc1	T, TT	I	R/R AML	China	NCT03473457
CD33, CD38, CD56, CD123, CLL-1, Muc1	T	I/II	AML	China	NCT03222674
NKG2D	T	I	AML, MDS-RAEB, Multiple Myeloma	USA	NCT02203825
NKG2D (NKR2)	T	I/II	R/R AML, Myeloma	USA + Belgium	NCT03018405

Abbreviations: CAR, chimeric antigen receptor; NCT, national clinical trial identifier; NK, natural killer cell; AML, acute myeloid leukemia; MDS, myelodysplastic syndrome; R/R, refractory/resistant; USA, United States of America; HSCT, hematopoietic stem cell transplantation; DCN, dendritic cell neoplasm; T, CAR-T cells; TT, double CAR-T cells; NK, CAR-natural killer cells; RAEB, refractory anemia with excess of blasts; NKG2D, natural killer group 2D antigen.

**Table 5:** Overview of strategies aimed at activating NK cells or applying (priming) NK cells or T cells as a therapeutic approach in patients with AML and CML.

Therapeutic Approach	Indication/Application
Standard therapies:	
Allogeneic hematopoietic stem cell transplantation (allo-HSCT)	Refractory or relapsed (R/R) AML and R/R advanced CML
Donor lymphocyte infusion (DLI)	Post allo-HSCT R/R AML and R/R CML after successful cytoreduction
Injection of IL-2 and histamine	Non-M3 AML-maintenance therapy
Experimental therapies *:	
Infusion of NK cells and/or T cells	R/R AML ** or AML in MRD
Infusion of allogeneic NK cells and/or T cells after HSCT	Post allo-HSCT R/R AML or R/R CML after successful re-induction
Infusion of antibody-primed T and/or NK cells	R/R AML ** or AML in MRD
Infusion of cytokine-activated T cells and/or NK cells (CIK)	R/R AML ** or AML in MRD
Infusion of CAR-T cells	R/R AML ** or AML in MRD
Infusion of CAR-NK cells	R/R AML ** or AML in MRD
Infusion of CIK CAR cells	R/R AML ** or AML in MRD

* These therapies are currently being tested in preclinical studies and/or clinical trials in patients with AML and/or other advanced myeloid neoplasms. ** In most instances, cell-based immunotherapy is combined with a de-bulking approach (polychemotherapy, hypomethylating agent, or cytostatic drug). Abbreviations: R/R, refractory/resistant; NK cells, natural killer cells; IL-2, interleukin-2; HSCT, hematopoietic stem cell transplantation; allo-HSCT, allogeneic HSCT; CML, chronic myeloid leukemia; CAR, chimeric antigen receptor; MRD, minimal residual disease; CIK, cytokine-induced killer cells.

**Table 6:** Mechanisms of resistance of leukemic stem cells (LSC) against immunotherapies.

Mechanism	Possible Strategy to Overcome Resistance
Intrinsic resistance	Antibody-based targeting of LSC
LSC quiescence	Antibody-based targeting of LSC Priming LSC into the cell cycle
Expression of MDR	MDR-targeting drugs or CAR cells
Loss of cell surface targets	Mixtures of antibodies or CAR cells directed against two or more surface targets, drug combinations, or combination of drug therapy with HSCT
Immune checkpoint-induced LSC resistance	Checkpoint-targeting antibodies Checkpoint-targeting CAR cells BET/MYC-targeting drugs * JAK/STAT-targeting drugs
BM niche-related resistance	Niche cell-targeting drugs
Osteoblastic niche	BET/MYC-targeting drugs *
Vascular niche	Specific anti-angiogenic drugs
LSC retention in niche	Mobilizing drugs (plerixafor)
LSC hypermobilization	Mobilization blocker (e.g., gliptins)
General immunosuppression	Repeated T/NK cell infusion
Blocked immune cells	Bispecific antibodies against LSC and immune effector cells
Loss of CAR-T cells or CAR-NK cells	Repeated infusions of CAR cells
Development of blocking antibodies against CARs	Use of single domain scFvs Humanize the scFvs

* BET/MYC-targeting drugs can suppress cytokine-induced and oncogene-induced expression of PD-L1 on LSC in AML and CML as well as osteoblast-induced resistance. Abbreviations: LSC, leukemic stem cells; MDR, multi-drug resistance gene product; HSCT, hematopoietic stem cell transplantation; CAR, chimeric antigen receptor; NK cells; natural killer cells; scFvs, single chain variable fragments.

## Abbreviations

NBM	normal bone marrow
FceRII	Fc-epsilon receptor II
IL-2RA	interleukin-2 receptor alpha chain
DPPIV	dipeptidyl peptidase IV
IAP	integrin associated protein
NCAM	neural cell adhesion molecule
G-CSFR	granulocyte colony-stimulating factor receptor
PROM1	prominin-1
PD-L1	programmed cell death-ligand 1
CLL-1	C-type lectin-like molecule-1
IL-1RAP	interleukin-1 receptor accessory protein
n.c.	not yet clustered
LSC	leukemic stem cells
AML	acute myeloid leukemia
CT	chemotherapy
HSCT	hematopoietic stem cell transplantation
AML	acute myeloid leukemia
NCT	national clinical trial identifier
BiTE	bispecific T cell engagers
TriKE	tri-specific killer engager
DART	dual affinity retargeting antibody
CAR	chimeric antigen receptor
NK	natural killer cell
AML	acute myeloid leukemia
MDS	myelodysplastic syndrome
R/R	refractory/resistant
USA	United States of America
HSCT	hematopoietic stem cell transplantation
DCN	dendritic cell neoplasm
NK	killer cells
RAEB	refractory anemia with excess of blasts
NKG2D	natural killer group 2D antigen

## References

[1] Estey E., Döhner H. (2006). Acute myeloid leukaemia. Lancet.

[2] Goldman J.M. (2007). Advances in CML. Clin. Adv. Hematol. Oncol..

[3] Hehlmann R., Hochhaus A., Baccarani M. (2007). European LeukemiaNet. Chronic myeloid leukaemia. Lancet.

[4] Smith M.L., Hills R.K., Grimwade D. (2011). Independent prognostic variables in acute myeloid leukaemia. Blood Rev..

[5] Marcucci G., Haferlach T., Döhner H. (2011). Molecular genetics of adult acute myeloid leukemia: Prognostic and therapeutic implications. J. Clin. Oncol..

[6] Moarii M., Papaemmanuil E. (2017). Classification and risk assessment in AML: Integrating cytogenetics and molecular profiling. ASH Educ. Program. Book.

[7] Visani G., Loscocco F., Isidori A., Piccaluga P.P. (2018). Genetic profiling in acute myeloid leukemia: A path to predicting treatment outcome. Expert Rev. Hematol..

[8] Kayser S., Levis M.J. (2019). Clinical implications of molecular markers in acute myeloid leukemia. Eur. J. Haematol..

[9] Andreeff M., Konopleva M. (2002). Mechanisms of drug resistance in AML. Cancer Treat. Res..

[10] Goldman J.M. (2009). Treatment strategies for CML. Best Pract. Res. Clin. Haematol..

[11] Burnett A., Wetzler M., Löwenberg B. (2011). Therapeutic advances in acute myeloid leukemia. J. Clin. Oncol..

[12] Castelli G., Pelosi E., Testa U. (2016). Targeted therapies in the treatment of adult acute myeloid leukemias: Current status and future perspectives. Int. J. Hematol. Oncol..

[13] Soverini S., Mancini M., Bavaro L., Cavo M., Martinelli G. (2018). Chronic myeloid leukemia: The paradigm of targeting oncogenic tyrosine kinase signaling and counteracting resistance for successful cancer therapy. Mol. Cancer.

[14] Lapidot T., Sirard C., Vormoor J., Murdoch B., Hoang T., Caceres-Cortes J., Minden M., Paterson B., Caligiuri M.A., Dick J.E. (1994). A cell initiating human acute myeloid leukaemia after transplantation into SCID mice. Nature.

[15] Bonnet D., Dick J.E. (1997). Human acute myeloid leukemia is organized as a hierarchy that originates from a primitive hematopoietic cell. Nat. Med..

[16] Hope K.J., Jin L., Dick J.E. (2004). Acute myeloid leukemia originates from a hierarchy of leukemic stem cell classes that differ in self-renewal capacity. Nat. Immunol..

[17] Barnes D.J., Melo J.V. (2006). Primitive, quiescent and difficult to kill: The role of non-proliferating stem cells in chronic myeloid leukemia. Cell Cycle.

[18] Krause D.S., Van Etten R.A. (2007). Right on target: Eradicating leukemic stem cells. Trends Mol. Med..

[19] Copland M. (2009). Chronic myelogenous leukemia stem cells: What’s new?. Curr. Hematol. Malig. Rep..

[20] Kavalerchik E., Goff D., Jamieson C.H. (2008). Chronic myeloid leukemia stem cells. J. Clin. Oncol..

[21] Essers M.A., Trumpp A. (2010). Targeting leukemic stem cells by breaking their dormancy. Mol. Oncol..

[22] Valent P. (2011). Targeting of leukemia-initiating cells to develop curative drug therapies: Straightforward but nontrivial concept. Curr. Cancer Drug Targets.

[23] Taussig D.C., Miraki-Moud F., Anjos-Afonso F., Pearce D.J., Allen K., Ridler C., Lillington D., Oakervee H., Cavenagh J., Agrawal S.G. (2008). Anti-CD38 antibody-mediated clearance of human repopulating cells masks the heterogeneity of leukemia-initiating cells. Blood.

[24] Taussig D.C., Vargaftig J., Miraki-Moud F., Griessinger E., Sharrock K., Luke T., Lillington D., Oakervee H., Cavenagh J., Agrawal S.G. (2010). Leukemia-initiating cells from some acute myeloid leukemia patients with mutated nucleophosmin reside in the CD34(-) fraction. Blood.

[25] Lemoli R.M., Salvestrini V., Bianchi E., Bertolini F., Fogli M., Amabile M., Tafuri A., Salati S., Zini R., Testoni N. (2009). Molecular and functional analysis of the stem cell compartment of chronic myelogenous leukemia reveals the presence of a CD34- cell population with intrinsic resistance to imatinib. Blood.

[26] Van Rhenen A., van Dongen G.A., Kelder A., Rombouts E.J., Feller N., Moshaver B., Stigter-van Walsum M., Zweegman S., Ossenkoppele G.J., Jan Schuurhuis G. (2007). The novel AML stem cell associated antigen CLL-1 aids in discrimination between normal and leukemic stem cells. Blood.

[27] Schulenburg A., Brämswig K., Herrmann H., Karlic H., Mirkina I., Hubmann R., Laffer S., Marian B., Shehata M., Krepler C. (2010). Neoplastic stem cells: current concepts and clinical perspectives. Crit. Rev. Oncol. Hematol..

[28] Järås M., Johnels P., Hansen N., Agerstam H., Tsapogas P., Rissler M., Lassen C., Olofsson T., Bjerrum O.W., Richter J. (2010). Isolation and killing of candidate chronic myeloid leukemia stem cells by antibody targeting of IL-1 receptor accessory protein. Proc. Natl. Acad. Sci. USA.

[29] Herrmann H., Sadovnik I., Cerny-Reiterer S., Rülicke T., Stefanzl G., Willmann M., Hoermann G., Bilban M., Blatt K., Herndlhofer S. (2014). Dipeptidylpeptidase IV (CD26) defines leukemic stem cells (LSC) in chronic myeloid leukemia. Blood.

[30] Eisterer W., Jiang X., Christ O., Glimm H., Lee K.H., Pang E., Lambie K., Shaw G., Holyoake T.L., Petzer A.L. (2005). Different subsets of primary chronic myeloid leukemia stem cells engraft immunodeficient mice and produce a model of the human disease. Leukemia.

[31] Jamieson C.H., Ailles L.E., Dylla S.J., Muijtjens M., Jones C., Zehnder J.L., Gotlib J., Li K., Manz M.G., Keating A. (2004). Granulocyte-macrophage progenitors as candidate leukemic stem cells in blast-crisis CML. N. Engl. J. Med..

[32] Blatt K., Menzl I., Eisenwort G., Cerny-Reiterer S., Herrmann H., Herndlhofer S., Stefanzl G., Sadovnik I., Berger D., Keller A. (2018). Phenotyping and Target Expression Profiling of CD34+/CD38- and CD34+/CD38+ Stem- and Progenitor cells in Acute Lymphoblastic Leukemia. Neoplasia.

[33] De Boer B., Prick J., Pruis M.G., Keane P., Imperato M.R., Jaques J., Brouwers-Vos A.Z., Hogeling S.M., Woolthuis C.M., Nijk M.T. (2018). Prospective Isolation and Characterization of Genetically and Functionally Distinct AML Subclones. Cancer Cell.

[34] Jordan C.T., Upchurch D., Szilvassy S.J., Guzman M.L., Howard D.S., Pettigrew A.L., Meyerrose T., Rossi R., Grimes B., Rizzieri D.A. (2000). The interleukin-3 receptor alpha chain is a unique marker for human acute myelogenous leukemia stem cells. Leukemia.

[35] Florian S., Sonneck K., Hauswirth A.W., Krauth M.T., Schernthaner G.H., Sperr W.R., Valent P. (2006). Detection of molecular targets on the surface of CD34+/CD38—Stem cells in various myeloid malignancies. Leuk. Lymphoma.

[36] Hauswirth A.W., Florian S., Printz D., Sotlar K., Krauth M.T., Fritsch G., Schernthaner G.H., Wacheck V., Selzer E., Sperr W.R. (2007). Expression of the target receptor CD33 in CD34+/CD38-/CD123+ AML stem cells. Eur. J. Clin. Investig..

[37] Hosen N., Park C.Y., Tatsumi N., Oji Y., Sugiyama H., Gramatzki M., Krensky A.M., Weissman I.L. (2007). CD96 is a leukemic stem cell-specific marker in human acute myeloid leukemia. Proc. Natl. Acad. Sci. USA.

[38] Majeti R., Chao M.P., Alizadeh A.A., Pang W.W., Jaiswal S., Gibbs K.D., van Rooijen N., Weissman I.L. (2009). CD47 is an adverse prognostic factor and therapeutic antibody target on human acute myeloid leukemia stem cells. Cell.

[39] Kersten B., Valkering M., Wouters R., van Amerongen R., Hanekamp D., Kwidama Z., Valk P., Ossenkoppele G., Zeijlemaker W., Kaspers G. (2016). CD45RA, a specific marker for leukaemia stem cell sub-populations in acute myeloid leukaemia. Br. J. Haematol..

[40] Cheng Y., Jia M., Chen Y., Zhao H., Luo Z., Tang Y. (2016). Re-evaluation of various molecular targets located on CD34+CD38-Lin- leukemia stem cells and other cell subsets in pediatric acute myeloid leukemia. Oncol. Lett..

[41] Sutherland H.J., Blair A., Zapf R.W. (1996). Characterization of a hierarchy in human acute myeloid leukemia progenitor cells. Blood.

[42] Saito Y., Kitamura H., Hijikata A., Tomizawa-Murasawa M., Tanaka S., Takagi S., Uchida N., Suzuki N., Sone A., Najima Y. (2010). Identification of therapeutic targets for quiescent, chemotherapy-resistant human leukemia stem cells. Sci. Transl. Med..

[43] Sperr W.R., Hauswirth A.W., Florian S., Ohler L., Geissler K., Valent P. (2004). Human leukaemic stem cells: A novel target of therapy. Eur. J. Clin. Investig..

[44] Iwasaki M., Liedtke M., Gentles A.J., Cleary M.L. (2015). CD93 Marks a Non-Quiescent Human Leukemia Stem Cell Population and Is Required for Development of MLL-Rearranged Acute Myeloid Leukemia. Cell Stem Cell.

[45] Hanekamp D., Cloos J., Schuurhuis G.J. (2017). Leukemic stem cells: Identification and clinical application. Int. J. Hematol..

[46] Blatt K., Herrmann H., Hoermann G., Willmann M., Cerny-Reiterer S., Sadovnik I., Herndlhofer S., Streubel B., Rabitsch W., Sperr W.R. (2014). Identification of Campath-1 (CD52) as novel drug target in neoplastic stem cells in 5q-patients with MDS and AML. Clin. Cancer Res..

[47] Blair A., Hogge D.E., Ailles L.E., Lansdorp P.M., Sutherland H.J. (1997). Lack of expression of Thy-1 (CD90) on acute myeloid leukemia cells with long-term proliferative ability in vitro and in vivo. Blood.

[48] Valent P., Sadovnik I., Ráčil Z., Herrmann H., Blatt K., Cerny-Reiterer S., Eisenwort G., Lion T., Holyoake T., Mayer J. (2014). DPPIV (CD26) as a novel stem cell marker in Ph+ chronic myeloid leukaemia. Eur. J. Clin. Investig..

[49] Sadovnik I., Hoelbl-Kovacic A., Herrmann H., Eisenwort G., Cerny-Reiterer S., Warsch W., Hoermann G., Greiner G., Blatt K., Peter B. (2016). Identification of CD25 as STAT5-Dependent Growth Regulator of Leukemic Stem Cells in Ph+ CML. Clin. Cancer Res..

[50] Warfvinge R., Geironson L., Sommarin M.N.E., Lang S., Karlsson C., Roschupkina T., Stenke L., Stentoft J., Olsson-Strömberg U., Hjorth-Hansen H. (2017). Single-cell molecular analysis defines therapy response and immunophenotype of stem cell subpopulations in CML. Blood.

[51] Culen M., Borsky M., Nemethova V., Razga F., Smejkal J., Jurcek T., Dvorakova D., Zackova D., Weinbergerova B., Semerad L. (2016). Quantitative assessment of the CD26+ leukemic stem cell compartment in chronic myeloid leukemia: patient-subgroups, prognostic impact, and technical aspects. Oncotarget.

[52] Gerber J.M., Gucwa J.L., Esopi D., Gurel M., Haffner M.C., Vala M., Nelson W.G., Jones R.J., Yegnasubramanian S. (2013). Genome-wide comparison of the transcriptomes of highly enriched normal and chronic myeloid leukemia stem and progenitor cell populations. Oncotarget.

[53] Janssen J.J., Deenik W., Smolders K.G., van Kuijk B.J., Pouwels W., Kelder A., Cornelissen J.J., Schuurhuis G.J., Ossenkoppele G.J. (2012). Residual normal stem cells can be detected in newly diagnosed chronic myeloid leukemia patients by a new flow cytometric approach and predict for optimal response to imatinib. Leukemia.

[54] Herrmann H., Cerny-Reiterer S., Gleixner K.V., Blatt K., Herndlhofer S., Rabitsch W., Jäger E., Mitterbauer-Hohendanner G., Streubel B., Selzer E. (2012). CD34+/CD38- stem cells in chronic myeloid leukemia express Siglec-3 (CD33) and are responsive to the CD33-targeting drug gemtuzumab/ozogamicin. Haematologica.

[55] Nievergall E., Ramshaw H.S., Yong A.S., Biondo M., Busfield S.J., Vairo G., Lopez A.F., Hughes T.P., White D.L., Hiwase D.K. (2014). Monoclonal antibody targeting of IL-3 receptor α with CSL362 effectively depletes CML progenitor and stem cells. Blood.

[56] Gerber J.M., Qin L., Kowalski J., Smith B.D., Griffin C.A., Vala M.S., Collector M.I., Perkins B., Zahurak M., Matsui W. (2011). Characterization of chronic myeloid leukemia stem cells. Am. J. Hematol..

[57] Testa U., Riccioni R., Militi S., Coccia E., Stellacci E., Samoggia P., Latagliata R., Mariani G., Rossini A., Battistini A. (2002). Elevated expression of IL-3Ralpha in acute myelogenous leukemia is associated with enhanced blast proliferation, increased cellularity, and poor prognosis. Blood.

[58] Yalcintepe L., Frankel A.E., Hogge D.E. (2006). Expression of interleukin-3 receptor subunits on defined subpopulations of acute myeloid leukemia blasts predicts the cytotoxicity of diphtheria toxin interleukin-3 fusion protein against malignant progenitors that engraft in immunodeficient mice. Blood.

[59] Lanza F., Castagnari B., Rigolin G., Moretti S., Latorraca A., Ferrari L., Bardi A., Castoldi G. (1997). Flow cytometry measurement of GM-CSF receptors in acute leukemic blasts, and normal hemopoietic cells. Leukemia.

[60] Graf M., Hecht K., Reif S., Pelka-Fleischer R., Pfister K., Schmetzer H. (2004). Expression and prognostic value of hemopoietic cytokine receptors in acute myeloid leukemia (AML): Implications for future therapeutical strategies. Eur. J. Haematol..

[61] Hogg S.J., Vervoort S.J., Deswal S., Ott C.J., Li J., Cluse L.A., Beavis P.A., Darcy P.K., Martin B.P., Spencer A. (2017). BET-bromodomain inhibitors engage the host immune system and regulate expression of the immune checkpoint ligand PD-L1. Cell Rep..

[62] Williams P., Basu S., Garcia-Manero G., Hourigan C.S., Oetjen K.A., Cortes J.E., Ravandi F., Jabbour E.J., Al-Hamal Z., Konopleva M. (2019). The distribution of T-cell subsets and the expression of immune checkpoint receptors and ligands in patients with newly diagnosed and relapsed acute myeloid leukemia. Cancer.

[63] Christiansson L., Söderlund S., Svensson E., Mustjoki S., Bengtsson M., Simonsson B., Olsson-Strömberg U., Loskog A.S. (2013). Increased level of myeloid-derived suppressor cells, programmed death receptor ligand 1/programmed death receptor 1, and soluble CD25 in Sokal high risk chronic myeloid leukemia. PLoS ONE.

[64] Majeti R. (2011). Monoclonal antibody therapy directed against human acute myeloid leukemia stem cells. Oncogene.

[65] Schulenburg A., Blatt K., Cerny-Reiterer S., Sadovnik I., Herrmann H., Marian B., Grunt T.W., Zielinski C.C., Valent P. (2015). Cancer stem cells in basic science and in translational oncology: can we translate into clinical application?. J. Hematol. Oncol..

[66] Pollyea D.A., Jordan C.T. (2017). Therapeutic targeting of acute myeloid leukemia stem cells. Blood.

[67] Bernstein I.D. (2000). Monoclonal antibodies to the myeloid stem cells: Therapeutic implications of CMA-676, a humanized anti-CD33 antibody calicheamicin conjugate. Leukemia.

[68] Bernstein I.D. (2002). CD33 as a target for selective ablation of acute myeloid leukemia. Clin. Lymphoma.

[69] Candoni A., Martinelli G., Toffoletti E., Chiarvesio A., Tiribelli M., Malagola M., Piccaluga P.P., Michelutti A., Simeone E., Damiani D. (2008). Gemtuzumab-ozogamicin in combination with fludarabine, cytarabine, idarubicin (FLAI-GO) as induction therapy in CD33-positive AML patients younger than 65 years. Leuk. Res..

[70] Satwani P., Bhatia M., Garvin J.H., George D., Dela Cruz F., Le Gall J., Jin Z., Schwartz J., Duffy D., van de Ven C. (2012). A Phase I study of gemtuzumab ozogamicin (GO) in combination with busulfan and cyclophosphamide (Bu/Cy) and allogeneic stem cell transplantation in children with poor-risk CD33+ AML: A new targeted immunochemotherapy myeloablative conditioning (MAC) regimen. Biol. Blood Marrow Transplant..

[71] Pelosi E., Castelli G., Testa U. (2015). Targeting LSCs through membrane antigens selectively or preferentially expressed on these cells. Blood Cells Mol. Dis..

[72] Walter R.B., Appelbaum F.R., Estey E.H., Bernstein I.D. (2012). Acute myeloid leukemia stem cells and CD33-targeted immunotherapy. Blood.

[73] Petersdorf S.H., Kopecky K.J., Slovak M., Willman C., Nevill T., Brandwein J., Larson R.A., Erba H.P., Stiff P.J., Stuart R.K. (2013). A phase 3 study of gemtuzumab ozogamicin during induction and postconsolidation therapy in younger patients with acute myeloid leukemia. Blood.

[74] Burnett A.K., Hills R.K., Milligan D., Kjeldsen L., Kell J., Russell N.H., Yin J.A., Hunter A., Goldstone A.H., Wheatley K. (2011). Identification of patients with acute myeloblastic leukemia who benefit from the addition of gemtuzumab ozogamicin: Results of the MRC AML15 trial. J. Clin. Oncol..

[75] Lichtenegger F.S., Krupka C., Köhnke T., Subklewe M. (2015). Immunotherapy for acute myeloid leukemia. Semin. Hematol..

[76] Hills R.K., Castaigne S., Appelbaum F.R., Delaunay J., Petersdorf S., Othus M., Estey E.H., Dombret H., Chevret S., Ifrah N. (2014). Addition of gemtuzumab ozogamicin to induction chemotherapy in adult patients with acute myeloid leukemia: A meta-analysis of individual patient data from randomized controlled trials. Lancet Oncol..

[77] Amadori S., Suciu S., Selleslag D., Aversa F., Gaidano G., Musso M., Annino L., Venditti A., Voso M.T., Mazzone C. (2016). Gemtuzumab ozogamicin versus best supportive care in older patients with newly diagnosed acute myeloid leukemia unsuitable for intensive chemotherapy: Results of the randomized Phase III EORTC-GIMEMA AML-19 trial. J. Clin. Oncol..

[78] Pollard J.A., Loken M., Gerbing R.B., Raimondi S.C., Hirsch B.A., Aplenc R., Bernstein I.D., Gamis A.S., Alonzo T.A., Meshinchi S. (2016). CD33 expression and its associated with gemtuzumab ozogamicin response: Results from the randomized Phase III children’s oncology group trial AAML0531. J. Clin. Oncol..

[79] Jen E.Y., Ko C.W., Lee J.E., Del Valle P.L., Aydanian A., Jewell C., Norsworthy K.J., Przepiorka D., Nie L., Liu J. (2018). FDA approval: Gemtuzumab ozogamicin for the treatment of adults with newly diagnosed CD33-positive acute myeloid leukemia. Clin. Cancer Res..

[80] Kung Sutherland M.S., Walter R.B., Jeffrey S.C., Burke P.J., Yu C., Kostner H., Stone I., Ryan M.C., Sussman D., Lyon R.P. (2013). SGN–CD33A: A novel targeting antibody–drug conjugate using a pyrrolobenzodiazepine dimer is active in models of drug-resistant AML. Blood.

[81] Stein E.M., Tallman M.S. (2016). Emerging therapeutic drugs for AML. Blood.

[82] Stein E.M., Walter R.B., Erba H.P., Fathi A.T., Advani A.S., Lancet J.E., Ravandi F., Kovacsovics T., DeAngelo D.J., Bixby D. (2018). A phase 1 trial of vadastuximab talirine as monotherapy in patients with CD33-positive acute myeloid leukemia. Blood.

[83] Walter R.B. (2018). Investigational CD33-targeted therapeutics for acute myeloid leukemia. Expert Opin. Investig. Drugs.

[84] Frankel A.E., McCubrey J.A., Miller M.S., Delatte S., Ramage J., Kiser M., Kucera G.L., Alexander R.L., Beran M., Tagge E.P. (2000). Diphteria toxin fused to human interleukin-3 is toxic to blasts from patients with myeloid leukemias. Leukemia.

[85] Testa U., Riccioni R., Biffoni M., Diverio D., Lo-Coco F., Foà R., Peschle C., Frankel A.E. (2005). Diphtheria toxin fused to variant human interleukin-3 induces cytotoxicity of blasts from patients with acute myeloid leukemia according to the level of interleukin-3 receptor expression. Blood.

[86] Cohen K.A., Liu T.F., Cline J.M., Wagner J.D., Hall P.D., Frankel A.E. (2004). Toxicology and pharmacokinetics of DT388IL3, a fusion protein consisting of a truncated diphtheria toxin (DT388) linked to human interleukin 3 (IL3) in cynomolgus monkeys. Leuk. Lymphoma.

[87] Hogge D.E., Yalcintepe L., Wong S.H., Gerhard B., Frankel A.E. (2006). Variant diphteria toxin-interleukin-3 fusion proteins with increased receptor affinity have enhanced cytotoxicity against acute myeloid leukemia progenitors. Clin. Cancer Res..

[88] Frankel A.E., Konopleva M., Hogge D., Rizzieri D., Brooks C., Cirrito T., Kornblau S.M., Borthakur G., Bivins C., Garcia-Manero G. (2013). Activity and tolerability of SL-401, a targeted therapy directed to the interleukin-3 receptor on cancer stem cells and tumor bulk, as a single agent in patients with advanced hematologic malignancies. J. Clin. Oncol..

[89] Frolova O., Benito J., Brooks C., Wang R.Y., Korchin B., Rowinsky E.K., Cortes J., Kantarjian H., Andreeff M., Frankel A.E. (2014). SL-401 and SL-501, targeted therapeutics directed at the interleukin-3 receptor, inhibit the growth of leukaemic cells and stem cells in advanced phase chronic myeloid leukaemia. Br. J. Haematol..

[90] Alkharabsheh O., Frankel A.E. (2019). Clinical Activity and Tolerability of SL-401 (Tagraxofusp): Recombinant Diphtheria Toxin and Interleukin-3 in Hematologic Malignancies. Biomedicines.

[91] Pemmaraju N., Lane A.A., Sweet K.L., Stein A.S., Vasu S., Blum W., Rizzieri D.A., Wang E.S., Duvic M., Sloan J.M. (2019). Tagraxofusp in Blastic Plasmacytoid Dendritic-Cell Neoplasm. N. Engl. J. Med..

[92] Syed Y.Y. (2019). Tagraxofusp: First global approval. Drugs.

[93] Jin L., Lee E.M., Ramshaw H.S., Busfield S.J., Peoppl A.G., Wilkinson L., Guthridge M.A., Thomas D., Barry E.F., Boyd A. (2009). Monoclonal antibody mediated targeting of CD123, IL-3 receptor alpha chain, eliminates human acute myeloid leukemia stem cells. Cell Stem Cell.

[94] Busfield S.J., Biondo M., Wong M., Ramshaw H.S., Lee E.M., Ghosh S., Braley H., Panousis C., Roberts A.W., He S.Z. (2014). Targeting of acute myeloid leukemia in vitro and in vivo with an anti-CD123 mAb engineered for optimal ADCC. Leukemia.

[95] He S.Z., Busfield S., Ritchie D.S., Hertzberg M.S., Durrant S., Lewis I.D., Marlton P., McLachlan A.J., Kerridge I., Bradstock K.F. (2015). Phase I study of the safety, pharmacokinetics and anti-leukemic activity of the anti-CD123 monoclonal antibody CSL360 in relapsed, refractory or high-risk acute myeloid leukemia. Leuk. Lymphoma.

[96] Jin L., Hope K.J., Zhai Q., Smadja-Joffe F., Dick J.E. (2006). Targeting of CD44 eradicates human acute myeloid leukemic stem cells. Nat. Med..

[97] Zhang L.Z., Ding X., Li X.Y., Cen J.N., Chen Z.X. (2010). In vitro effects of anti-CD44 monoclonal antibody on the adhesion and migration of chronic myeloid leukemia stem cells. Zhonghua Xue Ye Xue Za Zhi.

[98] Liu J., Wang L., Zhao F., Tseng S., Narayanan C., Shura L., Willingham S., Howard M., Prohaska S., Volkmer J. (2015). Pre-clinical development of a humanized anti-CD47 antibody with anti-cancer therapeutic potential. PLoS ONE.

[99] Vey N., Delaunay J., Martinelli G., Fiedler W., Raffoux E., Prebet T., Gomez-Roca C., Papayannidis C., Kebenko M., Paschka P. (2016). Phase I clinical study of RG7356, an anti-CD44 humanized antibody, in patients with acute myeloid leukemia. Oncotarget.

[100] Krupka C., Lichtenegger F.S., Köhnke T., Bögeholz J., Bücklein V., Roiss M., Altmann T., Do T.U., Dusek R., Wilson K. (2017). Targeting CD157 in AML using a novel, Fc-engineered antibody construct. Oncotarget.

[101] Kong F., Gao F., Li H., Liu H., Zhang Y., Zheng R., Zhang Y., Chen J., Li X., Liu G. (2016). CD47: a potential immunotherapy target for eliminating cancer cells. Clin. Transl. Oncol..

[102] Liu Y., Bewersdorf J.P., Stahl M., Zeidan A.M. (2019). Immunotherapy in acute myeloid leukemia and myelodysplastic syndromes: The dawn of a new era?. Blood Rev..

[103] Matthews D.C., Appelbaum F.R., Eary J.F., Fisher D.R., Durack L.D., Hui T.E., Martin P.J., Mitchell D., Press O.W., Storb R. (1999). Phase I study of (131) I-anti-CD45 antibody plus cyclophosphamide and total body irradiation for advanced acute leukemia and myelodysplastic syndrome. Blood.

[104] Bunjes D., Buchmann I., Duncker C., Seitz U., Kotzerke J., Wiesneth M., Dohr D., Stefanic M., Buck A., Harsdorf S.V. (2001). Rhenium 188-labeled anti-CD66 (a, b, c, e) monoclonal antibody to intensify the conditioning regimen prior to stem cell transplantation for patients with high-risk acute myeloid leukemia or myelodysplastic syndrome: Results of a phase I-II study. Blood.

[105] Pagel J.M., Appelbaum F.R., Eary J.F., Rajendran J., Fisher D.R., Gooley T., Ruffner K., Nemecek E., Sickle E., Durack L. (2006). 131I-anti-CD45 antibody plus busulfan and cyclophosphamide before allogeneic hematopoietic cell transplantation for treatment of acute myeloid leukemia in first remission. Blood.

[106] Bodet-Milin C., Kraeber-Bodéré F., Eugène T., Guérard F., Gaschet J., Bailly C., Mougin M., Bourgeois M., Faivre-Chauvet A., Chérel M. (2016). Radioimmunotherapy for Treatment of Acute Leukemia. Semin. Nucl. Med..

[107] Masarova L., Kantarjian H., Garcia-Mannero G., Ravandi F., Sharma P., Daver N. (2017). Harnessing the immune system against leukemia: Monoclonal antibodies and checkpoint strategies for AML. Adv. Exp. Med. Biol..

[108] Postow M.A., Callahan M.K., Wolchok J.D. (2015). Immune Checkpoint Blockade in Cancer Therapy. J. Clin. Oncol..

[109] Constantinidou A., Alifieris C., Trafalis D.T. (2019). Targeting Programmed Cell Death-1 (PD-1) and Ligand (PD-L1): A new era in cancer active immunotherapy. Pharmacol. Ther..

[110] Haroun F., Solola S.A., Nassereddine S., Tabbara I. (2017). PD-1 signaling and inhibition in AML and MDS. Ann. Hematol..

[111] Boddu P., Kantarjian H., Garcia-Manero G., Allison J., Sharma P., Daver N. (2018). The emerging role of immune checkpoint based approaches in AML and MDS. Leuk. Lymphoma.

[112] Masarova L., Kantarjian H., Ravandi F., Sharma P., Garcia-Manero G., Daver N. (2018). Update on immunotherapy in AML and MDS: Monoclonal antibodies and checkpoint inhibitors paving the road for clinical practice. Adv. Exp. Med. Biol..

[113] Giannopoulos K. (2019). Targeting Immune Signaling Checkpoints in Acute Myeloid Leukemia. J. Clin. Med..

[114] Daver N., Garcia-Manero G., Basu S., Boddu P.C., Alfayez M., Cortes J.E., Konopleva M., Ravandi-Kashani F., Jabbour E., Kadia T. (2019). Efficacy, Safety, and Biomarkers of response to azacitidine and nivolumab in relapsed/refractory acute myeloid leukemia: A nonrandomized, open-label, Phase II study. Cancer Discov..

[115] Berthon C., Driss V., Liu J., Kuranda K., Leleu X., Jouy N., Hetuin D., Quesnel B. (2010). In acute myeloid leukemia, B7-H1 (PD-L1) protection of blasts from cytotoxic T cells is induced by TLR ligands and interferon-gamma and can be reversed using MEK inhibitors. Cancer Immunol. Immunother..

[116] Krönig H., Kremmler L., Haller B., Englert C., Peschel C., Andreesen R., Blank C.U. (2014). Interferon-induced programmed death-ligand 1 (PD-L1/B7-H1) expression increases on human acute myeloid leukemia blast cells during treatment. Eur. J. Haematol..

[117] Dong P., Xiong Y., Yue J., Hanley S.J.B., Watari H. (2018). Tumor-Intrinsic PD-L1 Signaling in Cancer Initiation, Development and Treatment: Beyond Immune Evasion. Front. Oncol..

[118] Prestipino A., Emhardt A.J., Aumann K., O’Sullivan D., Gorantla S.P., Duquesne S., Melchinger W., Braun L., Vuckovic S., Boerries M. (2018). Oncogenic JAK2V617F causes PD-L1 expression, mediating immune escape in myeloproliferative neoplasms. Sci. Transl. Med..

[119] Yang H., Bueso-Ramos C., DiNardo C., Estecio M.R., Davanlou M., Geng Q.R., Fang Z., Nguyen M., Pierce S., Wei Y. (2014). Expression of PD-L1, PD-L2, PD-1 and CTLA4 in myelodysplastic syndromes is enhanced by treatment with hypomethylating agents. Leukemia.

[120] Zuber J., Shi J., Wang E., Rappaport A.R., Herrmann H., Sison E.A., Magoon D., Qi J., Blatt K., Wunderlich M. (2011). RNAi screen identifies Brd4 as a therapeutic target in acute myeloid leukaemia. Nature.

[121] Herrmann H., Blatt K., Shi J., Gleixner K.V., Cerny-Reiterer S., Müllauer L., Vakoc C.R., Sperr W.R., Horny H.P., Bradner J.E. (2012). Small-molecule inhibition of BRD4 as a new potent approach to eliminate leukemic stem- and progenitor cells in acute myeloid leukemia AML. Oncotarget.

[122] Davids M.S., Kim H.T., Bachireddy P., Costello C., Liguori R., Savell A., Lukez A.P., Avigan D., Chen Y.B., McSweeney P. (2016). Leukemia and lymphoma society blood cancer research partnership. Ipilimumab for patients with relapse after allogeneic transplantation. N. Engl. J. Med..

[123] Holderried T.A.W., Fraccaroli A., Schumacher M., Heine A., Brossart P., Stelljes M., Klobuch S., Kröger N., Apostolova P., Finke J. (2019). The role of checkpoint blockade after allogeneic stem cell transplantation in diseases other than Hodgkin’s Lymphoma. Bone Marrow Transplant..

[124] Kikushige Y., Miyamoto T., Yuda J., Jabbarzadeh-Tabrizi S., Shima T., Takayanagi S., Niiro H., Yurino A., Miyawaki K., Takenaka K. (2015). A TIM-3/Gal-9 autocrine stimulatory loop drives self-renewal of human myeloid leukemia stem cells and leukemic progression. Cell Stem Cell.

[125] Dama P., Tang M., Fulton N., Kline J., Liu H. (2019). Gal9/Tim-3 expression level is higher in AML patients who fail chemotherapy. J. Immunother. Cancer.

[126] Noviello M., Manfredi F., Ruggiero E., Perini T., Oliveira G., Cortesi F., De Simone P., Toffalori C., Gambacorta V., Greco R. (2019). Bone marrow central memory and memory stem T-cell exhaustion in AML patients relapsing after HSCT. Nat. Commun..

[127] Wolf E., Hofmeister R., Kufer P., Schlereth B., Baeuerle P.A. (2005). BiTEs: Bispecific antibody constructs with unique anti-tumor activity. Drug Discov. Today.

[128] Huehls A.M., Coupet T.A., Sentman C.L. (2015). Bispecific T-cell engagers for cancer immunotherapy. Immunol. Cell Biol..

[129] Schürch C.M. (2018). Therapeutic Antibodies for myeloid neoplasms-current developments and future directions. Front. Oncol..

[130] Guy D.G., Uy G.L. (2018). Bispecific Antibodies for the treatment of acute myeloid leukemia. Curr. Hematol. Malig. Rep..

[131] Wilke A.C., Gökbuget N. (2017). Clinical applications and safety evaluation of the new CD19 specific T-cell engager antibody construct blinatumomab. Expert Opin. Drug Saf..

[132] Ribera J.M. (2017). Efficacy and safety of bispecific T-cell engager blinatumomab and the potential to improve leukemia-free survival in B-cell acute lymphoblastic leukemia. Expert Rev. Hematol..

[133] Burt R., Warcel D., Fielding A.K. (2019). Blinatumomab, a bispecific B-cell and T-cell engaging antibody, in the treatment of B-cell malignancies. Hum. Vaccin. Immunother..

[134] Curran E., Stock W. (2019). Taking a “BiTE out of ALL”: Blinatumomab approval for MRD-positive ALL. Blood.

[135] Krupka C., Kufer P., Kischel R., Zugmaier G., Bögeholz J., Köhnke T., Lichtenegger F.S., Schneider S., Metzeler K.H., Fiegl M. (2014). CD33 target validation and sustained depletion of AML blasts in long-term cultures by the bispecific T-cell-engaging antibody AMG 330. Blood.

[136] Friedrich M., Henn A., Raum T., Bajtus M., Matthes K., Hendrich L., Wahl J., Hoffmann P., Kischel R., Kvesic M. (2014). Preclinical characterization of AMG 330, a CD3/CD33-bispecific T-cell-engaging antibody with potential for treatment of acute myelogenous leukemia. Mol. Cancer Ther..

[137] Laszlo G.S., Gudgeon C.J., Harrington K.H., Dell’Aringa J., Newhall K.J., Means G.D., Sinclair A.M., Kischel R., Frankel S.R., Walter R.B. (2014). Cellular determinants for preclinical activity of a novel CD33/CD3 bispecific T-cell engager (BiTE) antibody, AMG 330, against human AML. Blood.

[138] Stamova S., Cartellieri M., Feldmann A., Arndt C., Koristka S., Bartsch H., Bippes C.C., Wehner R., Schmitz M., von Bonin M. (2011). Unexpected recombinations in single chain bispecific anti-CD3-anti-CD33 antibodies can be avoided by a novel linker module. Mol. Immunol..

[139] Uy G., Godwin J., Rettig M., Vey N., Foster M., Arellano M., Rizzieri D., Topp M., Huls G., Lowenberg B. (2017). Preliminary results of a phase 1 study of flotetuzumab, a CD123 × CD3 bispecific Dart^®^ protein, in patients with relapsed/refractory acute myeloid leukemia and myelodysplastic syndrome. Blood.

[140] Gaudet F., Nemeth J.F., McDaid R., Li Y., Harman B., Millar H., Teplyakov A., Wheeler J., Luo J., Tam S. (2016). Development of a CD123 × CD3 bispecific antibody (JNJ-63709178) for the treatment of acute myeloid leukemia (AML). Blood.

[141] Chu S.Y., Pong E., Chen H., Phung S., Chan E.W., Endo N.A., Rashid R., Bonzon C., Leung I.W.L., Muchhal U.S. (2014). Immunotherapy with long-lived anti-CD123 × anti-CD3 bispecific antibodies stimulates potent T cell-mediated killing of human AML cell lines and of CD123+ cells in monkeys: a potential therapy for acute myelogenous leukemia. Blood.

[142] Van Loo P.F., Doornbos R., Dolstra H., Shamsili S., Bakker L. (2015). Preclinical evaluation of MCLA117, a CLEC12AxCD3 bispecific antibody efficiently targeting a novel leukemic stem cell associated antigen in AML. Blood.

[143] Krupka C., Kufer P., Kischel R., Zugmaier G., Lichtenegger F.S., Köhnke T., Vick B., Jeremias I., Metzeler K.H., Altmann T. (2016). Blockade of the PD-1/PD-L1 axis augments lysis of AML cells by the CD33/CD3 BiTE antibody construct AMG 330: Reversing a T-cell-induced immune escape mechanism. Leukemia.

[144] Al-Hussaini M., Rettig M.P., Ritchey J.K., Karpova D., Uy G.L., Eissenberg L.G., Gao F., Eades W.C., Bonvini E., Chichili G.R. (2016). Targeting CD123 in acute myeloid leukemia using a T-cell-directed dual-affinity retargeting platform. Blood.

[145] Leong S.R., Sukumaran S., Hristopoulos M., Totpal K., Stainton S., Lu E., Wong A., Tam L., Newman R., Vuillemenot B.R. (2017). An anti-CD3/anti-CLL-1 bispecific antibody for the treatment of acute myeloid leukemia. Blood.

[146] Hoseini S.S., Cheung N.K. (2017). Acute myeloid leukemia targets for bispecific antibodies. Blood Cancer J..

[147] Hoseini S.S., Guo H., Wu Z., Hatano M.N., Cheung N.V. (2018). A potent tetravalent T-cell-engaging bispecific antibody against CD33 in acute myeloid leukemia. Blood Adv..

[148] Bartels L., de Jong G., Gillissen M.A., Yasuda E., Kattler V., Bru C., Fatmawati C., van Hal-van Veen S.E., Cercel M.G., Moiset G. (2019). A chemo-enzymatically linked bispecific antibody retargets T cells to a sialylated epitope on CD43 in acute myeloid leukemia. Cancer Res..

[149] Casucci M., Nicolis di Robilant B., Falcone L., Camisa B., Norelli M., Genovese P., Gentner B., Gullotta F., Ponzoni M., Bernardi M. (2013). Cd44v6-targeted t cells mediate potent antitumor effects against acute myeloid leukemia and multiple myeloma. Blood.

[150] Wang Q.S., Wang Y., Lv H.Y., Han Q.W., Fan H., Guo B., Wang L.L., Han W.D. (2015). Treatment of CD33-directed chimeric antigen receptor-modified T cells in one patient with relapsed and refractory acute myeloid leukemia. Mol. Ther..

[151] Kenderian S.S., Ruella M., Shestova O., Klichinsky M., Aikawa V., Morrissette J.J., Scholler J., Song D., Porter D.L., Carroll M. (2015). CD33-specific chimeric antigen receptor t cells exhibit potent preclinical activity against human acute myeloid leukemia. Leukemia.

[152] Chien C.D., Sauter C.T., Ishii K., Nguyen S.M., Shen F., Tasian S.K., Chen W., Dimitrov D.S., Fry T.J. (2016). Preclinical development of flt3-redirected chimeric antigen receptor t cell immunotherapy for acute myeloid leukemia. Blood.

[153] Wang Y., Xu Y., Li S., Liu J., Xing Y., Xing H., Tian Z., Tang K., Rao Q., Wang M. (2018). Targeting flt3 in acute myeloid leukemia using ligand-based chimeric antigen receptor-engineered t cells. J. Hematol. Oncol..

[154] Laborda E., Mazagova M., Shao S., Wang X., Quirino H., Woods A.K., Hampton E.N., Rodgers D.T., Kim C.H., Schultz P.G. (2017). Development of a chimeric antigen receptor targeting c-type lectin-like molecule-1 for human acute myeloid leukemia. Int. J. Mol. Sci..

[155] Tashiro H., Sauer T., Shum T., Parikh K., Mamonkin M., Omer B., Rouce R.H., Lulla P., Rooney C.M., Gottschalk S. (2017). Treatment of acute myeloid leukemia with t cells expressing chimeric antigen receptors directed to c-type lectin-like molecule 1. Mol. Ther. J. Am. Soc. Gene Ther..

[156] Wang J., Chen S., Xiao W., Li W., Wang L., Yang S., Wang W., Xu L., Liao S., Liu W. (2018). CAR-T cells targeting CLL-1 as an approach to treat acute myeloid leukemia. J. Hematol. Oncol..

[157] Fan M., Li M., Gao L., Geng S., Wang J., Wang Y., Yan Z., Yu L. (2017). Chimeric antigen receptors for adoptive T cell therapy in acute myeloid leukemia. J. Hematol. Oncol..

[158] Hofmann S., Schubert M.L., Wang L., He B., Neuber B., Dreger P., Müller-Tidow C., Schmitt M. (2019). Chimeric Antigen Receptor (CAR) T Cell Therapy in Acute Myeloid Leukemia (AML). J. Clin. Med..

[159] Ritchie D.S., Neeson P.J., Khot A., Peinert S., Tai T., Tainton K., Chen K., Shin M., Wall D.M., Hönemann D. (2013). Persistence and efficacy of second generation CAR T cell against the LeY antigen in acute myeloid leukemia. Mol. Ther..

[160] Warda W., Larosa F., Neto Da Rocha M., Trad R., Deconinck E., Fajloun Z., Faure C., Caillot D., Moldovan M., Valmary-Degano S. (2019). CML hematopoietic stem cells expressing IL1RAP can be targeted by chimeric antigen receptor-engineered T cells. Cancer Res..

[161] Kottaridis P.D., North J., Tsirogianni M., Marden C., Samuel E.R., Jide-Banwo S., Grace S., Lowdell M.W. (2015). Two-stage priming of allogeneic natural killer cells for the treatment of patients with acute myeloid leukemia: A Phase I trial. PLoS ONE.

[162] Ruggeri L., Capanni M., Urbani E., Perruccio K., Shlomchik W.D., Tosti A., Posati S., Rogaia D., Frassoni F., Aversa F. (2002). Effectiveness of donor natural killer cell alloreactivity in mismatched hematopoietic transplants. Science.

[163] Lee D.A., Denman C.J., Rondon G., Woodworth G., Chen J., Fisher T., Kaur I., Fernandez-Vina M., Cao K., Ciurea S. (2016). Haploidentical natural killer cells infused before allogeneic stem cell transplantation for myeloid malignancies: A Phase I trial. Biol. Blood Marrow Transplant..

[164] Shaffer B.C., Le Luduec J.B., Forlenza C., Jakubowski A.A., Perales M.A., Young J.W., Hsu K.C. (2016). Phase II study of haploidentical natural killer cell infusion for treatment of relapsed or persistent myeloid malignancies following allogeneic hematopoietic cell transplantation. Biol. Blood Marrow Transplant..

[165] Curti A., Ruggeri L., Parisi S., Bontadini A., Dan E., Motta M.R., Rizzi S., Trabanelli S., Ocadlikova D., Lecciso M. (2016). Larger size of donor alloreactive NK cell repertoire correlates with better response to NK cell immunotherapy in elderly acute myeloid leukemia patients. Clin. Cancer Res..

[166] Björklund A.T., Clancy T., Goodridge J.P., Béziat V., Schaffer M., Hovig E., Ljunggren H.G., Ljungman P.T., Malmberg K.J. (2016). Naive donor NK cell repertoires associated with less leukemia relapse after allogeneic hematopoietic stem cell transplantation. J. Immunol..

[167] Berrien-Elliott M.M., Wagner J.A., Fehniger T.A. (2015). Human cytokine-induced memory-like natural killer cells. J. Innate Immun..

[168] Romee R., Rosario M., Berrien-Elliott M.M., Wagner J.A., Jewell B.A., Schappe T., Leong J.W., Abdel-Latif S., Schneider S.E., Willey S. (2016). Cytokine-induced memory-like natural killer cells exhibit enhanced responses against myeloid leukemia. Sci. Transl. Med..

[169] Przespolewski A., Szeles A., Wang E.S. (2018). Advances in immunotherapy for acute myeloid leukemia. Future Oncol..

[170] Hansrivijit P., Gale R.P., Barrett J., Ciurea S.O. (2019). Cellular therapy for acute myeloid Leukemia—Current status and future prospects. Blood Rev..

[171] Koerner S.P., Andre M.C., Leibold J.S., Kousis P.C., Kübler A., Pal M., Haen S.P., Bühring H.J., Grosse-Hovest L., Jung G. (2016). An Fc-optimized CD133 antibody for induction of NK cell reactivity against myeloid leukemia. Leukemia.

[172] Ruggeri L., Urbani E., Andre P., Mancusi A., Tosti A., Topini F., Bléry M., Animobono L., Romagné F., Wagtmann N. (2016). Effects of anti-NKG2A antibody administration on leukemia and normal hematopoietic cells. Haematologica.

[173] Klingemann H. (2014). Are natural killer cells superior CAR drivers?. Oncoimmunology.

[174] Introna M., Borleri G., Conti E., Franceschetti M., Barbui A.M., Broady R., Dander E., Gaipa G., D’Amico G., Biagi E. (2007). Repeated infusions of donor-derived cytokine-induced killer cells in patients relapsing after allogeneic stem cell transplantation: A Phase I study. Haematologica.

[175] Guo Y., Han W. (2015). Cytokine-induced killer (CIK) cells: From basic research to clinical translation. Chin. J. Cancer.

[176] Wang Y., Bo J., Dai H.R., Lu X.C., Lv H.Y., Yang B., Wang T., Han W.D. (2013). CIK cells from recurrent or refractory AML patients can be efficiently expanded in vitro and used for reduction of leukemic blasts in vivo. Exp. Hematol..

[177] Rettinger E., Huenecke S., Bonig H., Merker M., Jarisch A., Soerensen J., Willasch A., Bug G., Schulz A., Klingebiel T. (2016). Interleukin-15-activated cytokine-induced killer cells may sustain remission in leukemia patients after allogeneic stem cell transplantation: Feasibility, safety and first insights on efficacy. Haematologica.

[178] Tettamanti S., Marin V., Pizzitola I., Magnani C.F., Giordano Attianese G.M., Cribioli E., Maltese F., Galimberti S., Lopez A.F., Biondi A. (2013). Targeting of acute myeloid leukaemia by cytokine-induced killer cells redirected with a novel CD123-specific chimeric antigen receptor. Br. J. Haematol..

[179] Mandelli F., Vignetti M., Tosti S., Andrizzi C., Foa R., Meloni G. (1993). Interleukin 2 treatment in acute myelogenous leukemia. Stem Cells.

[180] Bergmann L., Heil G., Kolbe K., Lengfelder E., Puzicha E., Martin H., Lohmeyer J., Mitrou P.S., Hoelzer D. (1995). Interleukin-2 bolus infusion as late consolidation therapy in 2nd remission of acute myeloblastic leukemia. Leuk. Lymphoma.

[181] Blaise D., Attal M., Pico J.L., Reiffers J., Stoppa A.M., Bellanger C., Molina L., Nedellec G., Vernant J.P., Legros M. (1997). The use of a sequential high dose recombinant interleukin 2 regimen after autologous bone marrow transplantation does not improve the disease free survival of patients with acute leukemia transplanted in first complete remission. Leuk. Lymphoma.

[182] Brune M., Hansson M., Mellqvist U.H., Hermodsson S., Hellstrand K. (1996). NK cell-mediated killing of AML blasts: Role of histamine, monocytes and reactive oxygen metabolites. Eur J Haematol..

[183] Hellstrand K., Mellqvist U.H., Wallhult E., Carneskog J., Kimby E., Celsing F., Brune M. (1997). Histamine and interleukin-2 in acute myelogenous leukemia. Leuk. Lymphoma.

[184] Brune M., Castaigne S., Catalano J., Gehlsen K., Ho A.D., Hofmann W.K., Hogge D.E., Nilsson B., Or R., Romero A.I. (2006). Improved leukemia-free survival after postconsolidation immunotherapy with histamine dihydrochloride and interleukin-2 in acute myeloid leukemia: Results of a randomized phase 3 trial. Blood.

[185] Romero A.I., Thorén F.B., Aurelius J., Askarieh G., Brune M., Hellstrand K. (2009). Post-consolidation immunotherapy with histamine dihydrochloride and interleukin-2 in AML. Scand. J. Immunol..

[186] Nilsson M.S., Hallner A., Brune M., Nilsson S., Thorén F.B., Martner A., Hellstrand K. (2019). Immunotherapy with HDC/IL-2 may be clinically efficacious in acute myeloid leukemia of normal karyotype. Hum. Vaccines Immunother..

[187] Cuapio A., Post M., Cerny-Reiterer S., Gleixner K.V., Stefanzl G., Basilio J., Herndlhofer S., Sperr W.R., Brons N.H., Casanova E. (2016). Maintenance therapy with histamine plus IL-2 induces a striking expansion of two CD56bright NK cell subpopulations in patients with acute myeloid leukemia and supports their activation. Oncotarget.

[188] Sander F.E., Nilsson M., Rydström A., Aurelius J., Riise R.E., Movitz C., Bernson E., Kiffin R., Ståhlberg A., Brune M. (2017). Role of regulatory T cells in acute myeloid leukemia patients undergoing relapse-preventive immunotherapy. Cancer Immunol. Immunother..

[189] Nair R.R., Tolentino J., Hazlehurst L.A. (2010). The bone marrow microenvironment as a sanctuary for minimal residual disease in CML. Biochem. Pharmacol..

[190] Shafat M.S., Gnaneswaran B., Bowles K.M., Rushworth S.A. (2017). The bone marrow microenvironment—Home of the leukemic blasts. Blood Rev..

[191] Wang A., Zhong H. (2018). Roles of the bone marrow niche in hematopoiesis, leukemogenesis, and chemotherapy resistance in acute myeloid leukemia. Hematology.

[192] Martín-Henao G.A., Quiroga R., Sureda A., González J.R., Moreno V., García J. (2000). L-selectin expression is low on CD34+ cells from patients with chronic myeloid leukemia and interferon-a up-regulates this expression. Haematologica.

[193] Jongen-Lavrencic M., Salesse S., Delwel R., Verfaillie C.M. (2005). BCR/ABL-mediated downregulation of genes implicated in cell adhesion and motility leads to impaired migration toward CCR7 ligands CCL19 and CCL21 in primary BCR/ABL-positive cells. Leukemia.

[194] Ponnusamy K., Kohrs N., Ptasinska A., Assi S.A., Herold T., Hiddemann W., Lausen J., Bonifer C., Henschler R., Wichmann C. (2015). RUNX1/ETO blocks selectin-mediated adhesion via epigenetic silencing of PSGL-1. Oncogenesis.

[195] Uy G.L., Rettig M.P., Stone R.M., Konopleva M.Y., Andreeff M., McFarland K., Shannon W., Fletcher T.R., Reineck T., Eades W. (2017). A phase 1/2 study of chemosensitization with plerixafor plus G-CSF in relapsed or refractory acute myeloid leukemia. Blood Cancer J..

[196] Martínez-Cuadrón D., Boluda B., Martínez P., Bergua J., Rodríguez-Veiga R., Esteve J., Vives S., Serrano J., Vidriales B., Salamero O. (2018). A phase I-II study of plerixafor in combination with fludarabine, idarubicin, cytarabine, and G-CSF (PLERIFLAG regimen) for the treatment of patients with the first early-relapsed or refractory acute myeloid leukemia. Ann. Hematol..

[197] Corces M.R., Chang H.Y., Majeti R. (2017). Preleukemic Hematopoietic Stem Cells in Human Acute Myeloid Leukemia. Front. Oncol..

[198] Valent P., Bonnet D., De Maria R., Lapidot T., Copland M., Melo J.V., Chomienne C., Ishikawa F., Schuringa J.J., Stassi G. (2012). Cancer stem cell definitions and terminology: The devil is in the details. Nat. Rev. Cancer.

[199] Ding L., Ley T.J., Larson D.E., Miller C.A., Koboldt D.C., Welch J.S., Ritchey J.K., Young M.A., Lamprecht T., McLellan M.D. (2012). Clonal evolution in relapsed acute myeloid leukaemia revealed by whole-genome sequencing. Nature.

[200] Valent P., Bonnet D., Wöhrer S., Andreeff M., Copland M., Chomienne C., Eaves C. (2013). Heterogeneity of neoplastic stem cells: Theoretical, functional, and clinical implications. Cancer Res..

[201] Holyoake T.L., Vetrie D. (2017). The chronic myeloid leukemia stem cell: stemming the tide of persistence. Blood.

[202] Jiang X., Zhao Y., Smith C., Gasparetto M., Turhan A., Eaves A., Eaves C. (2007). Chronic myeloid leukemia stem cells possess multiple unique features of resistance to BCR-ABL targeted therapies. Leukemia.

[203] Zhou H.S., Carter B.Z., Andreeff M. (2016). Bone marrow niche-mediated survival of leukemia stem cells in acute myeloid leukemia: Yin and Yang. Cancer Biol. Med..

[204] Dotti G., Gottschalk S., Savoldo B., Brenner M.K. (2014). Design and development of therapies using chimeric antigen receptor-expressing t cells. Immunol. Rev..

[205] Liao D., Wang M., Liao Y., Li J., Niu T. (2019). A review of efficacy and safety of checkpoint inhibitor for the treatment of acute myeloid leukemia. Front. Pharmacol..

